# Integration of Reconfigurable p‐Bit and 1R Crossbar Array for Memristive Probabilistic Computing

**DOI:** 10.1002/advs.76719

**Published:** 2026-07-23

**Authors:** Keunho Soh, Ji Eun Kim, Suk Yeop Chun, Su In Hwang, Byung Seok Kim, Young Jae Lee, Ho Won Jang, Jung Ho Yoon

**Affiliations:** ^1^ School of Advanced Materials Science and Engineering Sungkyunkwan University (SKKU) Suwon Republic of Korea; ^2^ Department of Materials Science and Engineering Research Institute of Advanced Materials Seoul National University Seoul Republic of Korea; ^3^ Electronic and Hybrid Materials Research Center Korea Institute of Science and Technology (KIST) Seoul Republic of Korea; ^4^ Department of Materials Science and Engineering Korea University Seoul Republic of Korea; ^5^ KU‐KIST Graduate School of Converging Science and Technology Korea University Seoul Republic of Korea; ^6^ Department of Semiconductor Convergence Engineering Sungkyunkwan University Suwon Republic of Korea

**Keywords:** combinatorial optimization, crossbar array, memristor, probabilistic computing, reversible logic

## Abstract

Probabilistic computing has emerged as an efficient paradigm for solving complex problems with high dimensionality and massive combinatorial search spaces. In particular, combinatorial optimization problems (COPs) can be mapped onto energy‐based models described by an interaction (*J*) matrix and bias (*h*) vector. In hardware implementations, these parameters can be physically encoded as conductance values in a resistive crossbar array (CBA), while probabilistic bits (p‐bits) provide a stochastic update of the energy state based on a weighted‐sum operation across the CBA. Here, we present a memristive probabilistic computing system that integrates volatile memristors as p‐bits and non‐volatile memristors organized in a selector‐less 1R CBA for representing the *J* matrix and *h* vector. The volatile memristors perform stochastic conductive filament formation and rupture to generate reconfigurable probabilistic outputs, while the non‐volatile memristors form a nanocluster‐based conductive path that enable reliable array operation without additional selector devices. The integrated system is experimentally validated through reversible AND and NAND gate operations in both forward and inverse modes. Furthermore, the scalability and generality of the proposed architecture were evaluated through simulation of a 2‐bit binary multiplier operation. This work presents a practical and scalable framework for fully hardware‐based probabilistic computing using memristive technologies.

## Graphical abstract

A memristive probabilistic computing system is demonstrated by integrating stochastic p‐bits based on volatile memristors with a 1R crossbar array encoding interaction weights. The system performs weighted‐sum operations across the array and updates p‐bits iteratively. Reversible logic gates and a binary multiplier are realized, showing scalable, reconfigurable hardware for combinatorial optimization.

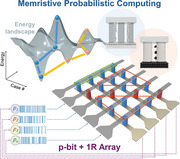

## 1 Introduction

Combinatorial optimization problems (COPs) arise in a wide range of scientific and engineering applications, where the objective is to identify optimal configurations from exponentially large discrete search spaces. Representative examples include scheduling, [1, 2] satisfiability, [3, 4] and graph optimization problems, [5, 6] all of which exhibit rapidly increasing computational complexity as system size grows. Conventional deterministic digital computing architectures are often inefficient for solving such problems, as they rely on exhaustive search or iterative numerical methods [7]. Probabilistic computing offers an alternative paradigm by treating randomness as a computational resource, enabling efficient exploration of complex energy landscapes and convergence toward the lowest energy solutions [8].

**FIGURE 1:**
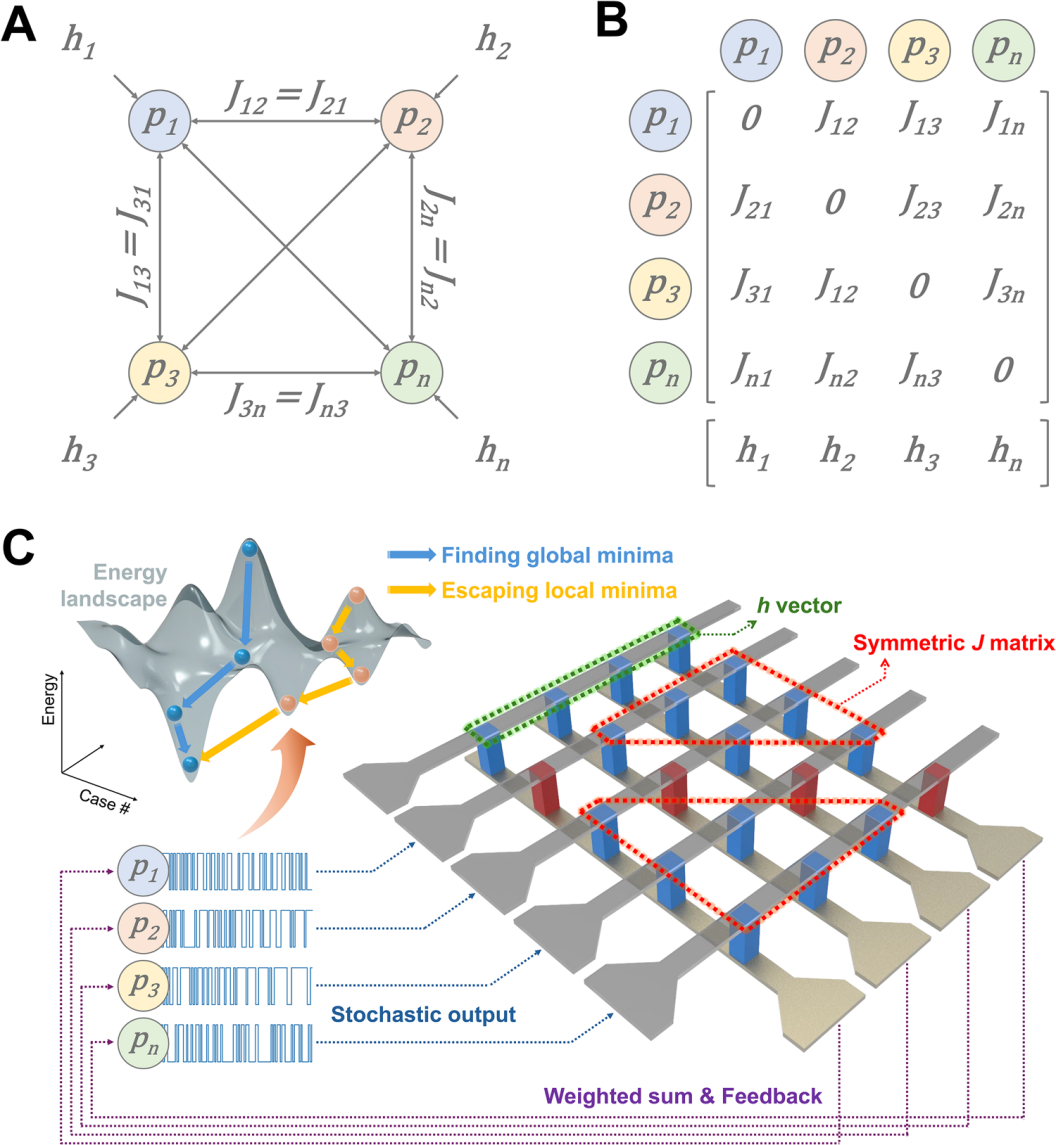
Conceptual framework of probabilistic computing and its hardware implementation. (A) Graph representation of an energy‐based model, where nodes denote p‐bits and edges represent pairwise interactions *J_ij_* with local biases *h_i_*. (B) Equivalent matrix formulation using the *J* matrix and *h* vector, defining the all‐to‐all connectivity and weighted‐sum computation for each p‐bit. (C) Hardware implementation using a CBA, where *J* and *h* are encoded as conductance states. Stochastic p‐bit outputs are applied as inputs, and the resulting currents perform parallel weighted‐sum operations and feedback, enabling convergence toward the global minimum.

Such problems can be formulated as energy minimization problems defined by pairwise interactions and local biases:

$$E=-\sum_{i<j}J_{ij}p_{i}p_{j}-\sum_{i}h_{i}p_{i}$$

where *p_i_* ∈ {0, 1} denotes the binary state of the *i*‐th variable, *J_ij_* represents the interaction between variables, and *h_i_* is a bias term. This energy function can be represented as a graph, where nodes correspond to variables and edges represent pairwise interactions (Figure 1A). In hardware probabilistic computing systems, each variable *p_i_* is mapped onto a corresponding probabilistic bit (p‐bit), which stochastically generates binary states. Solving the COP corresponds to finding the minimum of this energy function. Also, the update direction of each variable is determined from the negative partial derivative of the energy:

$$I_{i}=-\;\frac{\partial E}{\partial p_{i}}=\sum_{j}J_{ij}p_{j}+h_{i}$$

which takes the form of a weighted sum of all interacting variables and the local bias term. This formulation can be expressed equivalently in matrix form, where the symmetric J matrix encodes all pairwise couplings, yielding an all‐to‐all‐connected p‐bit network, as illustrated in Figure 1B. Accordingly, computing *I_i_* for all variables corresponds to a vector‐matrix multiplication (VMM), which must be performed repeatedly during iterative updates. This makes VMM a core computational bottleneck in probabilistic computing. Notably, resistive crossbar arrays (CBAs), widely used in neuromorphic and in‐memory computing, naturally perform VMM via Ohm's and Kirchhoff's laws, enabling parallel and analog weighted‐sum operations [9, 10]. Therefore, CBAs can be directly adopted to compute *I_i_* efficiently, offering significantly faster and more energy‐efficient operation compared to external processor‐based implementations of the partial derivative [11].

A conceptual hardware implementation is illustrated in Figure 1C, where the *J* matrix and *h* vector are encoded as conductance states within the CBA. Stochastic p‐bit outputs are applied as input voltages to the array, and the resulting currents summed across the CBA naturally perform the weighted‐sum operation. The resulting analog signals are fed back as inputs for subsequent updates. Through iterative weighted‐sum updates, the system progressively moves toward lower‐energy states. At the same time, the stochastic output characteristics of the p‐bits allow the system to escape local minima, enabling convergence toward the global minimum of the energy landscape. Based on this framework, hardware implementations of probabilistic computing systems require stochastic p‐bits that generate binary states with controllable probabilities, a crossbar array that encodes the *J* matrix and *h* vector as conductance states, and peripheral circuitry that performs signal scaling and feedback for iterative p‐bit updates.

Recently, various approaches have been explored to realize hardware p‐bits and stochastic computing elements, including magnetic tunnel junctions, [12, 13] ferroelectric tunnel junctions, [14] memristors, [15, 16, 17] memtransistors, [18, 19] and CMOS‐compatible transistors [20, 21]. In parallel, CBAs have been extensively developed as efficient hardware platforms for VMM, [22, 23, 24] owing to their high density and inherent capability for parallel weighted‐sum operations. However, most prior work has focused on either p‐bit implementation [25, 26, 27] or CBA‐based computation [28, 29, 30] independently. Consequently, a fully integrated hardware probabilistic computing system that couples stochastic p‐bits with CBA‐based weight representation and computation remains largely unexplored.

Ion‐motion‐mediated memristors based on the electrochemical metallization (ECM) mechanism provide a particularly attractive device platform for such systems, as their switching behavior can be rationally engineered by selecting mobile metal species that govern the formation and dissolution of conductive paths. When highly mobile cations are employed, continuous conductive filaments (CFs) are readily formed under an applied electric field. Because these highly mobile filaments are weakly stabilized and can rapidly dissolve or rupture after bias removal, such devices naturally exhibit volatile switching with cycle‐to‐cycle stochasticity, making them well‐suited for implementing p‐bits [31, 32]. On the other hand, when less mobile metal species with relatively large activation energy for ion diffusion, such as Ru, are introduced, conductive paths are not necessarily formed as continuous CFs but instead tend to evolve through spatially distributed metal nanoclusters [33, 34]. In this case, switching occurs via field‐driven redistribution of metal species and the gradual modulation of tunneling distances between neighboring clusters. This enables stable non‐volatile switching and analog conductance modulation, which are suitable for storing and tuning the weight parameters in CBAs, including the *J* matrix and *h* vector.

In this work, we propose and experimentally demonstrate a memristive probabilistic computing system that integrates ion‐motion‐mediated volatile memristors as p‐bits and non‐volatile cluster‐type memristors arranged in a selector‐less 1R CBA for encoding the *J* matrix and *h* vector. By interfacing these components with peripheral analog circuitry for signal scaling and differential amplification, we realize a complete hardware platform capable of performing probabilistic combinatorial optimization. The proposed system is experimentally validated through reversible logic gate operations (AND and NAND) in both forward and inverse modes, and its scalability is further demonstrated through simulations of a 2‐bit binary multiplier. These results indicate a practical and scalable framework toward fully hardware‐based probabilistic computing systems using memristive technologies.

## 2 Results and Discussion

### 2.1 Stochastic Volatile Memristor for p‐Bit Implementation

**FIGURE 2:**
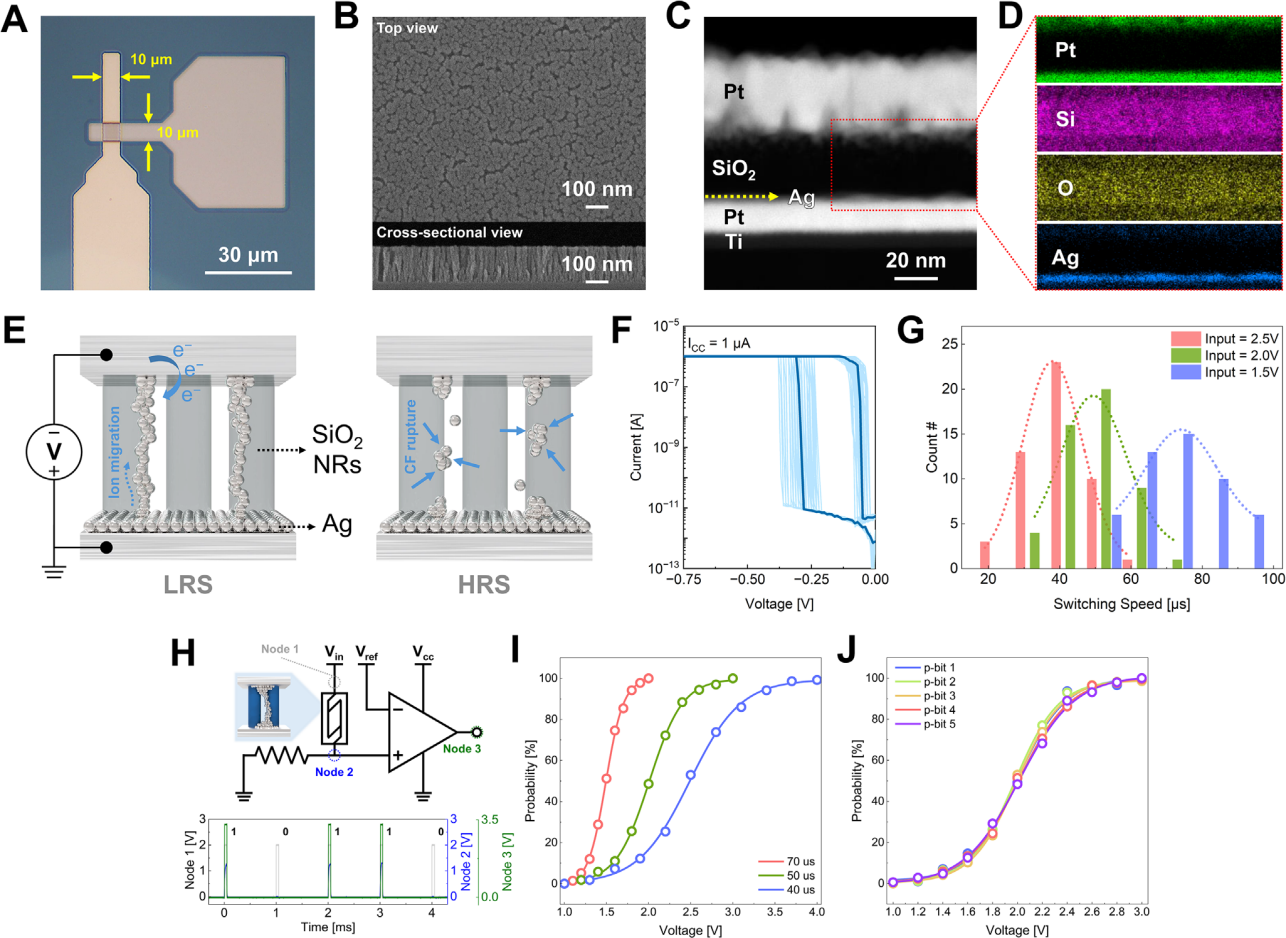
Device structure and stochastic switching characteristics of the volatile memristor and p‐bit implementation. (A) Top‐view optical image of the fabricated volatile memristor. (B) Top and cross‐sectional view SEM images of the porous NRs‐structured SiO_2_ film. (C) Cross‐sectional STEM image of the fabricated volatile memristor. (D) EDS elemental mapping of Pt, Si, O, and Ag. (E) Illustration of the ECM mechanism operating in the fabricated NRs‐based volatile memristor. (F) Typical *I–V* curves depicting the electrical resistive switching behavior of the fabricated volatile memristor. (G) Measured switching speed distribution depending on input pulse amplitude. (H) Schematic of a p‐bit circuit incorporating a volatile memristor, with monitored voltage signals at each node demonstrating pulse‐triggered switching behavior. (I) Output probability data depending on input pulse width measured by increasing the input pulse amplitude, fitted with a sigmoid curve characteristic of p‐bit. (J) Overlapped sigmoid curves measured from p‐bits constructed using five randomly selected volatile memristors.

A top‐view optical image of a volatile memristor used for a p‐bit implementation is shown in Figure 2A. It features a resistive switching area of 10 µm × 10 µm with Pt (30 nm)/SiO_2_ (30 nm)/Ag (5 nm)/Pt (15 nm)/Ti (5 nm) stacked structure. The SiO_2_ layer inserted between Pt and Ag was deposited as a nanorods‐based (NRs) porous film using the glancing‐angle deposition method (see Experimental Section for the fabrication process). The porosity of the SiO_2_ NRs layer was clearly observed in top‐ and cross‐sectional scanning electron microscopy (SEM) images, as shown in Figure 2B. Then, a cross‐sectional scanning transmission microscopy (STEM) image confirms that each layer is deposited to the intended thickness (Figure 2C), and Energy‐dispersive x‐ray spectroscopy (EDS) mapping further verifies the spatial distribution of Ag, Pt, Si, and O, confirming the integrity of the device stack (Figure 2D).

The fabricated volatile memristor was designed to perform abrupt resistive switching based on ECM mechanism [35, 36]. As illustrated in Figure 2E, the volatile memristor exhibits a high‐resistance state (HRS) by default due to the SiO_2_ NRs insulating layer. When a voltage bias exceeding the threshold voltage (V_th_) is applied to the volatile memristor, Ag cations are generated through electrochemical oxidation of the Ag bottom electrode (BE). Subsequently, positively charged Ag cations migrate toward the top electrode (TE) under the electric field applied across the volatile memristor. During ion migration, NR‐based morphology is advantageous because Ag cations preferentially migrate along NR surfaces, where the migration barrier is lower than in bulk SiO_2_ [37]. As a result, higher ion mobility and enhanced switching speed can be achieved. Afterward, Ag cations reach the TE, capture electrons, and are reduced to Ag atoms. The continuous accumulation of these reduced Ag atoms eventually forms CFs that bridge the TE and BE, thereby switching the volatile memristor to the low‐resistance state (LRS). When the voltage bias is removed, the volatile memristor returns to the HRS owing to the spontaneous rupture of CFs driven by surface energy minimization, thereby exhibiting volatile behavior. During the resistive switching process, multiple CFs form and rupture at random locations within the switching area. Thus, the complex interplay among CF location, the sequence of CF formation and rupture, and the bias‐dependent real‐time reinforcement or Joule‐heat‐assisted diffusion of CFs contributes to the switching speed of the volatile memristor, which is inherently impossible to define precisely [38].

Based on ion‐motion dynamics, stochastic electrical switching characteristics were evaluated using direct current (DC) sweeps and switching speed measurements. DC sweep measurements were conducted over 100 cycles from 0 to −0.75 V with a step size of 0.01 V under a compliance current (I_CC_) of 1 µA. The measured current–voltage (*I–V*) curves of the volatile memristor are shown in Figure 2F. The volatile memristor exhibited threshold switching behavior with an average V_th_ of 0.29 V and a standard deviation (σ) of 0.034 V, along with an ON/OFF current ratio of approximately 10^5^, indicating inherent stochasticity in the switching process. Switching speed measurements were subsequently performed using square pulses with amplitudes of 1.5, 2.0, and 2.5 V and a fixed pulse width of 500 µs, with 50 switching cycles measured at each amplitude. The average switching speeds were 71.69, 50.41, and 37.83 µs for pulse amplitudes of 1.5, 2.0, and 2.5 V, respectively, with corresponding σ of 12.08, 8.91, and 8.52 µs (Figure 2G). As the pulse amplitude increased, the average switching speed increased and the distribution narrowed. These results indicate that higher pulse amplitudes enhance switching speed, which is attributed to increased Ag^+^ ion generation and accelerated ion migration under stronger electric fields. Therefore, increasing the pulse amplitude at a fixed pulse width increases the probability that the volatile memristor switches to the LRS. Notably, increasing the pulse width at a fixed amplitude similarly enhances the switching probability, as Ag cation migration continues while the electrical stimulus is maintained. Therefore, precise control of the HRS‐to‐LRS switching probability can be achieved by simultaneously modulating the amplitude and pulse width of the input signal.

By utilizing the stochastic switching characteristics shown in Figure 2G, a p‐bit incorporating a volatile memristor was constructed to convert the device switching probability into a refined output. Figure 2H presents a schematic of the p‐bit, comprising a volatile memristor, a resistor, and an operational amplifier (OP‐AMP), along with the voltage outputs at each node. When an input pulse (V_in_) is applied to Node 1, most of the voltage initially drops across the volatile memristor in the HRS due to voltage division. If the volatile memristor switches to the LRS before the end of the input pulse, the voltage distribution changes, resulting in a larger voltage drop across the series resistor and generating a signal at Node 2. This signal is then applied to the non‐inverting input of the OP‐AMP. When the signal exceeds the reference voltage (V_ref_) at the inverting input, the OP‐AMP produces a binary output with an amplitude of V_CC_ at Node 3. The output probability characteristics of the p‐bit are shown in Figure 2I. To evaluate the probabilistic output behavior, a 1 kHz pulse train was applied to the volatile memristor, and the probability of output generation during 500 pulses was measured under pulse widths of 40, 50, and 70 µs. The input amplitude varied from 1 to 4 V in increments of 0.3 V, from 1 to 3 V in increments of 0.2 V, and from 1 to 2 V in increments of 0.1 V, respectively. At a fixed pulse width, the output probability increased with input amplitude (Figure S1). These probability distributions were well fitted by sigmoid curves, representing dynamic ranges of p‐bit. Longer pulse widths enable operation at lower voltage amplitudes with a reduced input range, but require finer amplitude control, whereas shorter pulse widths allow a wider voltage range with relaxed amplitude resolution at the cost of higher operating voltages. This trade‐off enables the sigmoid characteristics of the p‐bit to be reconfigured via pulse width, thereby allowing adaptation to the specifications of the peripheral circuitry that generates V_in_. The stability of the probabilistic response was further verified by comparing the sigmoid characteristics before and after 10^5^ repeated stochastic switching cycles, which revealed negligible change in the output probability distribution (Figure S2). Additionally, sigmoid curves obtained under a 50 µs pulse width condition from five p‐bits constructed using five randomly selected volatile memristors exhibited a uniform trend in probabilistic output characteristics, indicating low device‐to‐device variation (Figure 2J and Figure S3). The corresponding 50% switching points averaged 2.00014 V with a maximum deviation of ±18.8 mV, indicating low device‐to‐device variation. A detailed discussion of the variation and its implication for tolerance to input perturbations is provided in Note S1. Additional autocorrelation analysis of stochastic bit streams is also provided in Figure S2, confirming memoryless stochastic behavior across multiple p‐bits.

### 2.2 Cluster‐Type Non‐Volatile 1R Crossbar Array

**FIGURE 3:**
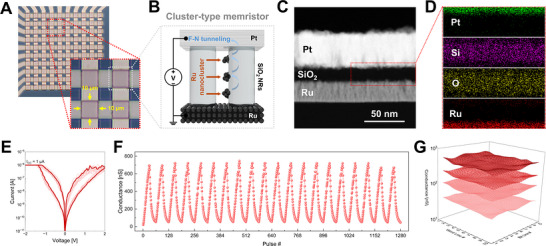
Device structure and analog switching characteristics of the cluster‐type non‐volatile 1R CBA. (A) Top‐view optical image of the fabricated 10 × 10 1R CBA. (B) Illustration of the cluster‐type conductive path within the fabricated NRs‐based non‐volatile memristor junction. (C) Cross‐sectional STEM image of the fabricated non‐volatile memristor. (D) EDS elemental mapping of Pt, Si, O, and Ru. (E) Typical *I–V* curves depicting the electrical resistive switching behavior of the fabricated non‐volatile memristor. (F) Conductance modulation performance and cycle endurance characteristic of the fabricated non‐volatile memristor. (G) Four distinct conductance levels observed across the 10 × 10 1R CBA.

The top‐view optical image of the well‐fabricated 10 × 10 selector‐less 1‐resistor crossbar array (1R CBA) used for *J* matrix and *h* vector implementation is shown in Figure 3A. Each junction between the word line (WL), serving as the TE, and the bit line (BL), serving as the BE, defines a resistive switching area of 10 µm × 10 µm and consists of a Pt (40 nm)/SiO_2_ NRs (20 nm)/Ru (25 nm) stacked structure. The SiO_2_ layer inserted between Pt and Ru was deposited using the same glancing‐angle deposition method employed for the volatile memristor described in Section 2.1. The fabricated non‐volatile memristor junction was designed to achieve gradual analog resistive switching based on Ru nanoclusters, as illustrated in Figure 3B. Indeed, distinct layers corresponding to Pt WL, Ru BL, and SiO_2_ NRs layers were confirmed in the cross‐sectional STEM image (Figure 3C) and EDS maps (Figure 3D).

Ru has been explored as a promising mobile species for non‐volatile memristor implementation because it exhibits a relatively large activation energy for diffusion and slower redox kinetics than highly mobile cations such as Ag or Cu. As a result, the formation of continuous CFs is suppressed, while nanocluster‐type conductive paths are preferentially developed, thereby enabling low‐current operation and good retention characteristics [33, 39]. In addition, the NRs structure of the SiO_2_ layer facilitates Ru cation migration even under low I_CC_ conditions owing to the locally reduced migration barrier along the NR surface. Thus, conductive paths composed of spatially distributed Ru nanoclusters can be formed within the SiO_2_ NR layer using only a limited amount of Ru species. Unlike abrupt CF formation and rupture in filamentary memristors, switching in this cluster‐type conductive path occurs through the gradual field‐driven redistribution of Ru species and the corresponding modulation of tunneling distances between adjacent nanoclusters, which act as tunneling sites. As a result, abrupt resistance transitions are suppressed, leading to stable non‐volatile switching with gradual, linear, and analog conductance modulation characteristics. Such characteristics are particularly advantageous for CBA‐based weight representation and tuning, as they can encode analog input parameters, such as the *J* matrix and *h* vector, that are interfaced with p‐bits to generate stochastic binary outputs. In probabilistic computing systems, these input amplitudes are determined by weighted sums obtained through VMM in the CBA. As the complexity of the system increases, the *J* matrix and *h* vector span a wider range of weights, requiring the resulting analog signals to be finely resolved within the dynamic range of the p‐bit sigmoid response. To ensure distinct probabilistic outputs, the VMM results must be accurately mapped to this operating range, which, in turn, requires stable multilevel conductance states in the CBA. In this regard, the gradual and linear conductance modulation of cluster‐type memristors provides a suitable platform for reliable analog weight representation and precise control of p‐bit inputs. Furthermore, the LRS formed by the Ru nanocluster‐based conductive path exhibits nonlinear conduction behavior owing to tunneling transport between adjacent nanoclusters. Fowler–Nordheim tunneling becomes dominant as the local electric field across the nanoscale gaps increases, reducing the effective potential barrier height and width and thereby inducing a nonlinear increase in current [40]. Such field‐dependent conduction suppresses leakage current through unselected or half‐selected cells at low bias, while allowing sufficient current flow through the selected cell at the read voltage. As a result, the nonlinear LRS provides intrinsic self‐rectification to mitigate sneak‐current paths, thereby enabling selector‐less CBA operation.

Accordingly, a representative *I–V* curve of the cluster‐type non‐volatile memristor is shown in Figure 3E. DC sweep measurements were performed from 0 to −2 V for the SET process (HRS to LRS) and from 0 to 2 V for the RESET process (LRS to HRS) with a step size of 0.05 V under a I_CC_ of 1 µA. During the SET process, the non‐volatile memristor reached the I_CC_ below −1.5 V, demonstrating low‐power switching capability. The non‐volatile memristor also exhibited low LRS‐current while maintaining gradual switching characteristics. In addition, an inherent *I–V* nonlinearity was observed in the low‐voltage region between −0.4 and 0.4 V, where the current was significantly suppressed. A quantitative analysis of the read margin and maximum achievable CBA size based on the measured *I–V* characteristics is provided in Note S2 and Figure S4. Figure 3F further demonstrates the conductance modulation characteristics of the non‐volatile memristor. Gradual SET (Potentiation) was achieved using 32 pulses of −2.5 V amplitude with a pulse width of 1 µs, whereas gradual RESET (Depression) was performed using 32 pulses of 2.5 V amplitude with the same pulse width. A read voltage of −0.25 V was used as an offset during conductance measurement. Over 20 repeated modulation cycles (corresponding to 1280 total pulse inputs), the non‐volatile memristor exhibited highly linear and symmetric conductance modulation, demonstrating its versatility and good endurance. The relatively high programming amplitude (±2.5 V) was intentionally selected during single‐device characterization to reveal a broad conductance‐tuning range. Additional measurements of the conductance modulation behavior under reduced programming amplitudes are provided in Figure S5. The programmed conductance states also exhibited stable retention under both room‐temperature and elevated‐temperature conditions (Figure S6), supporting reliable long‐term weight storage. Furthermore, a contour plot of the conductance modulation characteristics for all 100 junction memristors in the CBA is shown in Figure 3G. The non‐volatile memristors exhibited four representative, clearly distinguishable conductance levels, selected to demonstrate reliable and discrete programmability. This indicates that the fabricated 1R CBA is well‐suited for conductance‐based representation of the *J* matrix and *h* vector.

### 2.3 System‐Level Integration

To demonstrate the capability of the proposed memristive probabilistic computing system across different levels of complexity, Boolean operations and combinatorial logic were employed as representative test cases. Boolean logic underlies digital computation, and its reversible implementation enables both forward evaluation and inverse solution search within the same framework. The AND gate, as the simplest three‐node logic unit, serves as a minimal test case for evaluating probabilistic convergence, particularly in inverse operations, where multiple valid input combinations yield the same output. The NAND gate, being functionally complete, confirms that arbitrary logic functions can be implemented within the proposed architecture. Notably, this functionality can be achieved solely by programming the conductance states of the 1R CBA, without additional circuit elements. Furthermore, cascading structures can be readily constructed by assigning shared p‐bits across interconnected logic units, enabling scalable system integration [41]. Finally, a 2‐bit binary multiplier operation was investigated through simulation as a combinatorial logic example to demonstrate scalability and multifunctionality. When operated in reverse, the same system performs factorization, indicating that multiple combinatorial tasks can be executed within a single hardware framework defined by a given *J* matrix and *h* vector.

**FIGURE 4:**
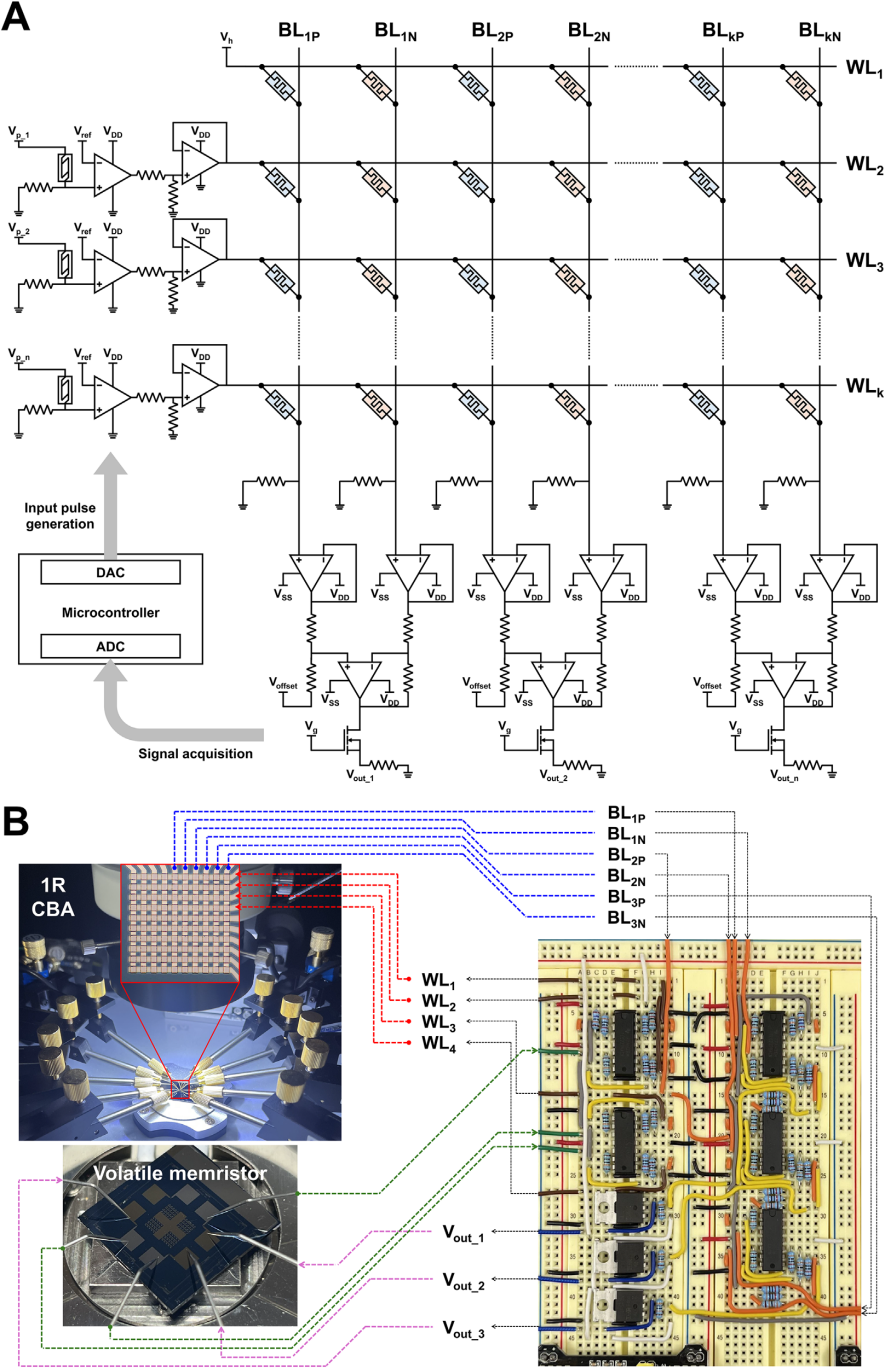
System‐level integration of the memristive probabilistic computing system. (A) Schematic of proposed memristive probabilistic computing system incorporating p‐bit, 1R CBA, and peripheral circuit. (B) Breadboard‐based peripheral circuits are integrated with the 1R CBA and volatile memristors loaded on a probe station. Overlaid annotations illustrate the electrical connections between the WLs and BLs of the CBA and the TEs and BEs of the volatile memristor.

The proposed schematic of a memristive probabilistic computing system is illustrated in Figure 4A. The *J* matrix and *h* vector corresponding to a target COP are programmed as conductance values in the 1R CBA. To represent both positive and negative weights, two adjacent columns are assigned to each weight, where odd‐numbered BLs store positive weights and even‐numbered BLs store negative weights [42].

The stochastic outputs of the p‐bits are applied to the WLs. To prevent unintended conductance variation in the CBA caused by direct p‐bit output application, a voltage‐divider node composed of series resistors and an OP‐AMP‐based buffer was inserted between the p‐bit output node and the WL input node. When a p‐bit generates an output pulse, the signal is scaled to an amplitude of 1 V through the voltage divider before being applied to the non‐inverting input of the buffer. Owing to the high input impedance of the buffer, the scaled signal is delivered to the WL without disturbing the voltage‐divider network.

WL_1_ corresponds to the *h* vector and is maintained at a continuous 1 V pulse, while the remaining WLs receive stochastic input signals from the p‐bits. The resulting currents are summed along each BL and converted into voltage signals through pull‐down resistors. To prevent interference in current summation from subsequent amplification stages, the converted voltage signals are first applied to the non‐inverting inputs of dedicated buffers connected to each BL. The outputs of these buffers are then fed into differential amplifiers configured for each adjacent column pair, which subtract the negative‐weight contribution from the positive‐weight contribution. When current flows only through the negative‐weight branch, the subtracted output signal can exhibit a negative voltage amplitude. To enable signal acquisition by the analog‐to‐digital converter, which is limited to positive input voltages, an offset voltage (V_offset_) is added to ensure that all signals remain positive. Subsequently, an NMOS switch removes the V_offset_ component from the differential amplifier output through gate voltage pulse (V_g_) control. Since WL_1_ is continuously held 1 V pulse, this signal is amplified above the NMOS V_th_ and utilized as the gate‐control signal. The final weighted‐sum signal is then acquired by the microcontroller through a pull‐down resistor connected to the NMOS source node. By precisely tuning the gain of the differential amplifiers, the full possible weighted‐sum signal range, spanning from the minimum to maximum weighted‐sum amplitudes, was adjusted to match the sigmoid‐fitted operating range of the p‐bit output under the 50 µs pulse width condition (1 to 3 V). As a result, the microcontroller can directly generate a V_p_n_ pulse with an amplitude identical to that of the acquired weighted‐sum signal, without requiring additional computation, thereby enabling efficient utilization in the subsequent iteration.

All peripheral circuits, excluding the volatile memristors and the 1R CBA, were fabricated on a breadboard. Figure 4B shows the assembled hardware implementation, including the peripheral circuitry and optical images of the 1R CBA and volatile memristors loaded on a probe station, along with a schematic overlay indicating the connections between the WLs, BLs, and the TEs and BEs of the volatile memristors. It should be noted that the present probabilistic computing platform was implemented using commercially available discrete peripheral electronics, including OP‐AMP, NMOS, and microcontroller‐assisted feedback control. Consequently, the measured system‐level area and energy characteristics are strongly influenced by the laboratory‐scale implementation and the use of discrete peripheral electronics. Nevertheless, the peripheral circuitry scales approximately linearly with the number of p‐bits, whereas the accessible probabilistic state space increases exponentially.

### 2.4 Probabilistic Computing Demonstrations

**FIGURE 5:**
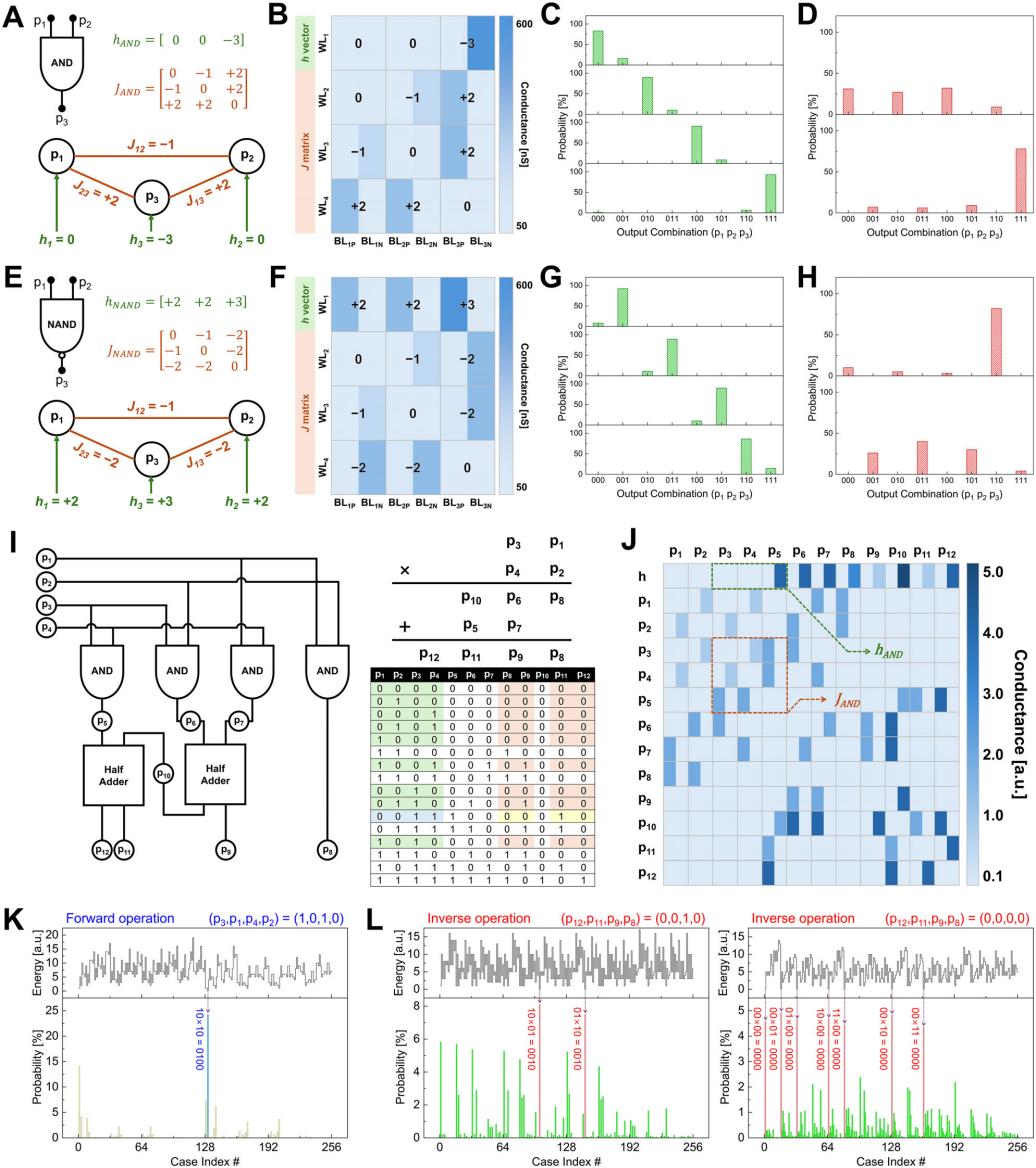
Reversible logic gate operations and scalability demonstration using the proposed memristive probabilistic computing system. (A) AND gate symbol, corresponding *J* matrix and *h* vector, and associated graph representation. (B) Conductance map programmed into a 6 × 4 region of the 1R CBA for AND gate implementation. (C) Forward operation results of the AND gate, showing dominant convergence to the correct output state (p_3_) for fixed inputs (p_1_, p_2_). (D) Inverse operation results of the AND gate, showing exploration of all valid input combinations for p_3_ = 0 and convergence to the unique valid input combination for p_3_ = 1. (E) NAND gate symbol, corresponding *J* matrix and *h* vector, and associated graph representation. (F) Reconfigured conductance map in the 1R CBA for NAND gate implementation. (G) Forward operation results of the NAND gate, confirming correct output behavior. (H) Inverse operation results of the NAND gate, demonstrating correct input‐state exploration and convergence. (I) Schematic of the 2‐bit binary multiplier and assigned p‐bit mapping for each input and output node, along with corresponding truth table. The two multiplicands are encoded by (p_3_, p_1_) and (p_4_, p_2_), while the product bits are represented by (p_12_, p_11_, p_9_, p_8_) from MSB to LSB. (J) Conductance map of the 2‐bit multiplier implemented by merging the *J* matrix and *h* vector of constituent AND gates and half adders. (K) Forward operation results of the 2‐bit multiplier with (p_3_, p_1_, p_4_, p_2_) = (1, 0, 1, 0), where the correct output state appears with the highest probability at the lowest energy level, confirming proper multiplication functionality. (L) Inverse operation results of the 2‐bit multiplier with (p_12_, p_11_, p_9_, p_8_) = (0, 0, 1, 0) and (p_12_, p_11_, p_9_, p_8_) = (0, 0, 0, 0), where multiple valid input states corresponding to lowest energy levels exhibit distinctly higher probabilities, confirming correct factorization behavior.

By integrating the peripheral circuit shown in Figure 4B with volatile memristors and the 1R CBA, reversible logic gate operations that serve as representative COPs were demonstrated. Although conventional logic gates generate output signals from given inputs, they can be modeled as COPs to identify valid output combinations for a given input and valid input combinations for a specified output [43]. Figure 5A shows the AND gate symbol, its corresponding *J* matrix and *h* vector, and the associated graph representation. It should be noted that, unlike spintronic‐device‐based probabilistic computing systems employing the Ising model with spin states *s_i_* ∈ {−1, 1}, [44] memristive p‐bits operate using Boolean variables *p_i_* ∈ {0, 1}, requiring quadratic unconstrained binary optimization‐based *J* matrix and *h* vector construction [45] (see Note S3 for the *J* matrix and *h* vector derivation). For a reversible AND gate implementation, three p‐bits were assigned: p_1_ and p_2_ as inputs, and p_3_ as the output. The conductance map programmed into a 6 × 4 region of the 1R CBA is shown in Figure 5B. To ensure probabilistic operation, the CBA‐generated local‐field values were calibrated to the experimentally measured sigmoid characteristic through the peripheral circuitry. In addition, transient signal propagation, including the effects of parasitic components and OP‐AMP bandwidth, was evaluated and confirmed to preserve distinguishable local‐field levels during operation (Figure S7 and Note S4).

When 100 stochastic iterations were collected, all eight possible state combinations (000–111) were observed, along with the monitored output signals for p_1_, p_2_, and p_3_ (Figure S8A). Among these, the four valid combinations of the AND gate (000, 010, 100, and 111) exhibited probabilities exceeding 20%, indicating that all valid states were frequently observed under unconstrained stochastic operation. For forward operation, in which V_p_1_ and V_p_2_ were fixed at either 1 or 3 V, the output p_3_ was dominantly obtained according to the AND logic. Specifically, when (p_1_, p_2_) = (0, 0), (0, 1), (1, 0), and (1, 1), the corresponding p_3_ output was predominantly observed as 0, 0, 0, and 1, respectively (Figure 5C), with the monitored stochastic output traces provided in Figure S9. For the inverse operation, in which V_p_3_ was fixed at either 1 or 3 V, Figure 5D shows that p_1_ and p_2_ explored all valid combinations when p_3_ = 0 and converged to the unique valid solution (p_1_, p_2_) = (1, 1) when p_3_ = 1. The corresponding stochastic output signals are shown in Figure S10.

The same procedure was repeated for a NAND gate to demonstrate the reconfigurability of the implemented probabilistic computing system. The NAND gate symbol, corresponding *J* matrix and *h* vector, and associated graph representation are shown in Figure 5E. The reconfigured conductance map programmed into the 1R CBA is shown in Figure 5F, while the forward and inverse operation results are presented in Figure 5G, H, respectively (see Figure S8B for the unconstrained stochastic distribution). In the unconstrained case, all eight possible states were observed, and the four valid NAND gate combinations (001, 011, 101, and 110) exhibited probabilities exceeding 20%, indicating frequent occurrence of valid states (Figure S8B). For forward operation, when (p_1_, p_2_) = (0, 0), (0, 1), (1, 0), and (1, 1) were fixed, the corresponding p_3_ output was predominantly observed as 1, 1, 1, and 0, respectively, consistent with NAND logic (Figure 5G), with the monitored stochastic output traces provided in Figure S11. For inverse operation, when p_3_ was fixed, p_1_ and p_2_ explored all valid combinations corresponding to the given output, confirming correct inverse functionality (Figure 5H), with the corresponding stochastic output signals shown in Figure S12. All measured results confirm the correct and reconfigurable logic gate functionality within the probabilistic computing framework. To further assess the practical efficiency of the proposed architecture, we estimated the energy consumption of the p‐bit, the 1R CBA read operation, and the demonstrated overall system. The estimated p‐bit update energy and average CBA read energy were approximately 102 pJ/update and 0.94 pJ/read, respectively, yielding a total system‐level energy of approximately 31.16 nJ for the 100‐iteration (Figure S13). A quantitative comparison with previously reported probabilistic computing implementations is provided in Table S1.

Finally, a 2‐bit binary multiplier modeled as a COP was simulated to assess the scalability of the proposed system (see the Experimental Section and Figures S14 and S15 for simulation details). The schematic of the 2‐bit multiplier and the assigned p‐bit mapping for each input/output node, along with its corresponding truth table, are provided in Figure 5I. The 2‐bit multiplier can be constructed from four AND gates and two half adders (HAs), with p‐bits assigned to all input and output nodes. In this configuration, the *J* matrix and *h* vector of the multiplier do not need to be derived independently. Instead, the multiplier can be implemented by merging the *J* matrix and *h* vector of the constituent AND gates and HAs [41, 46] (see Figure S16 for the HA *J* matrix and *h* vector). The conductance map based on the merged *J* matrix and *h* vector is shown in Figure 5J, where the representative contribution from an AND gate is indicated by a green dotted box for the *J* matrix and a brown dotted box for the *h* vector.

The simulated forward and inverse operation results are presented in Figure 5K, L, respectively, each consisting of an upper energy landscape showing all possible state energies and a lower plot showing the corresponding probability distribution. Although the full system consists of 12 p‐bits (4096 possible states), constraining four p‐bits for input or output reduces the solution space to 256 states, over which the energy landscape is evaluated. Throughout the simulation, the two multiplicands are represented by (p_3_, p_1_) and (p_4_, p_2_), respectively, where the first and second elements correspond to the most significant bit (MSB) and least significant bit (LSB), respectively. The product bits were interpreted in the order (p_12_, p_11_, p_9_, p_8_), where p_12_ and p_8_ correspond to the MSB and LSB, respectively. For the forward operation (Figure 5K), the input p‐bits are fixed at (p_3_, p_1_, p_4_, p_2_) = (1, 0, 1, 0) corresponding to the binary multiplication of 10 × 10. The correct output state (p_12_, p_11_, p_9_, p_8_) = (0, 1, 0, 0) exhibits the highest probability at the lowest energy level, confirming proper multiplication functionality. For the inverse operation (Figure 5L), the output p‐bits are constrained to specific values to evaluate factorization behavior. When (p_12_, p_11_, p_9_, p_8_) = (0, 0, 1, 0), the valid input combinations 10 × 01 and 01 × 10 are correctly identified with high probabilities. When (p_12_, p_11_, p_9_, p_8_) = (0, 0, 0, 0), all seven valid input combinations (00 × 00, 00 × 01, 01 × 00, 10 × 00, 00 × 10, 11 × 00, and 00 × 11) emerge with distinctly higher and nearly uniform probabilities, reflecting the equally valid lowest energy states.

Notably, inverse multiplier operation inherently functions as a factorizer, which is highly advantageous because implementing dedicated factorizer circuits in conventional CMOS‐based digital systems is typically area‐intensive [47]. Moreover, since integer factorization underpins widely deployed cryptographic protocols such as RSA [48], it serves as a practically relevant benchmark for evaluating specialized optimization hardware. Because the proposed system allows arbitrary reconfiguration of the *J* matrix and *h* vector depending on the target COP and supports adjustable p‐bit sigmoid fitting ranges, it represents a highly scalable, reconfigurable, and versatile probabilistic computing platform.

## 3 Conclusion

In this work, we have demonstrated a fully integrated memristor‐based probabilistic computing system that unifies stochastic p‐bits and analog weight representation within a single hardware platform. Ion‐motion‐mediated memristors based on the ECM mechanism were systematically engineered to exhibit distinct functionalities depending on the choice of mobile species. Volatile memristors employing highly mobile Ag^+^ cations enabled stochastic CF formation and rupture, providing intrinsic randomness and threshold switching behavior suitable for p‐bit implementation. In contrast, non‐volatile cluster‐type memristors based on less mobile Ru formed distributed nanocluster conduction paths, enabling stable, gradual, and linear conductance modulation for reliable encoding of the *J* matrix and *h* vector in a selector‐less 1R CBA.

By integrating these components with peripheral analog circuitry, including voltage scaling, buffering, and differential amplification, a complete hardware system for probabilistic computing was realized. The 1R CBA performs VMM through analog current summation, and the resulting signals are directly fed back to the p‐bits. This structure enables iterative weighted‐sum updates while maintaining stochastic state transitions, allowing the system to approach lower‐energy states without being trapped in local minima.

The functionality of the proposed system was experimentally validated through reversible Boolean logic operations, including AND and NAND gates, demonstrating both forward evaluation and inverse solution search within the same hardware. The reconfigurability of the system was achieved solely through conductance programming of the CBA, without modifying circuit topology. Furthermore, system scalability and generality were confirmed through simulations of a 2‐bit binary multiplier, where both multiplication and factorization operations were successfully demonstrated within a unified framework defined by a single *J* matrix and *h* vector.

Overall, the proposed architecture provides a hardware implementation of probabilistic computing that integrates stochastic p‐bits and in‐memory analog computation. The experimentally demonstrated AND and NAND gates, together with simulation‐based multiplier results, support its applicability to larger COPs and its use as a building block for scalable optimization hardware based on memristive technologies.

## 4 Experimental Section/Methods

### 4.1 Device and Array Fabrication

Volatile memristor with Pt/SiO_2_ NRs/Ag/Pt/Ti structure was fabricated on SiO_2_/Si substrates using standard photolithography and lift‐off techniques. The BE composed of Ag/Pt/Ti with thickness of 5 nm for Ag, followed by 15 nm for Pt and 5 nm for Ti was deposited through electron‐beam evaporation. Subsequently, an insulating SiO_2_ layer with thickness of 30 nm was also deposited on the BE using electron‐beam evaporation. A glancing angle of 60° was employed to promote self‐shadowing effects during SiO_2_ deposition, resulting in porous NRs structure. The Pt TE with thickness of 30 nm was then deposited using an electron‐beam evaporator at the same glancing angle of 60° and a rotation speed of 15 rpm. This deposition approach prevented unintended diffusion of Pt into the porous SiO_2_ NRs. Simultaneously, 1R CBA comprising 10 × 10 non‐volatile memristor cells with Pt/SiO_2_ NRs/Ru structure was fabricated on SiO_2_/Si substrates using photolithography and lift‐off techniques. The Ru BE with thickness of 25 nm was deposited through DC magnetron sputtering. Subsequently, an insulating SiO_2_ layer with thickness of 20 nm followed by the Pt TE with thickness of 40 nm was deposited through electron‐beam evaporation at the same glancing angle of 60° and a rotation speed of 15 rpm.

### 4.2 Device Characterization

Cross‐sectional imaging of the Pt/SiO_2_ NRs/Ag/Pt/Ti and Pt/SiO_2_/Ru structures were examined using TEM (JEM‐F200, JEOL) and SEM (JSM‐7600F, JEOL). An intuitive analysis was performed using EDS on the TEM sample prepared using a focused ion beam (Helios 5 UX, Thermo Fisher Scientific) for elemental analysis. Electrical switching behavior was measured under ambient conditions using a 4155A semiconductor parameter analyzer (HP), applying a bias to the TE with the BE grounded. Pulse measurements were performed using an AFG‐3102C function generator and a DSO‐X 3024T oscilloscope.

### 4.3 Circuit Implementation

Power supplying DC voltage was generated using AMS1117‐5.0 (Advanced Monolithic Systems) voltage regulator. The circuit component TLC2274 (Texas Instruments) for OP‐AMP and IRF540NPbF (Infineon Technologies) for NMOS were used. The acquisition of stochastic output signals from p‐bits, the weighted summation of voltage‐converted stochastic pulse outputs, and the generation of operating input voltages for p‐bits in the subsequent iteration were performed using an Arduino Due microcontroller based on the Atmel SAM3×8E ARM Cortex‐M3 CPU.

### 4.4 Probabilistic Computing Simulation

Virtual p‐bit implementation and stochastic output signal acquisition were conducted in a Python environment (PyCharm, JetBrains), whereas input and output signal generation were performed using the LTspice simulation platform (Analog Devices).

## Author Contributions

**Suk Yeop Chun**: data curation, formal analysis. **Ji Eun Kim**: formal analysis, data curation. **Young Jae Lee**: formal analysis, data curation. **Ho Won Jang**: supervision, writing – review and editing. **Byung Seok Kim**: formal analysis, data curation. **Jung Ho Yoon**: conceptualization, methodology, supervision, funding acquisition, project administration, writing – review and editing, resources. **Keunho Soh**: conceptualization, methodology, software, data curation, investigation, formal analysis, writing – original draft, validation, visualization. **Su In Hwang**: data curation, formal analysis.

## Funding

This research was supported by the National R&D Program through the National Research Foundation of Korea (NRF) and the Korea Basic Science Institute (KBSI), funded by the Ministry of Science and ICT (RS‐2024‐00406418, RS‐2024‐00403917, RS‐2025‐02215065, and RS‐2026‐25480979).

## Conflicts of Interest

The authors declare no conflict of interest.

## Supporting information




**Supporting File**: advs76719‐sup‐0001‐SuppMat.pdf.

## Data Availability

The data that support the findings of this study are available from the corresponding author upon reasonable request.
