# Supplementary material for: Integration of Reconfigurable p‐Bit and 1R Crossbar Array for Memristive Probabilistic Computing
Source: Adv Sci (Weinh). 2026 Jul 23:e76719. Online ahead of print. doi: 10.1002/advs.76719 (PMC13393278; doi:10.1002/advs.76719)
Supplement: Supplementary file 1 — Supporting File: advs76719‐sup‐0001‐SuppMat.pdf. [file ADVS-9999-e76719-s001.pdf]

Supporting Information

Integration of Reconfigurable p-Bit and 1R Crossbar Array for Memristive Probabilistic Computing

*Keunho Soh[1,2], Ji Eun Kim[3,4], Suk Yeop Chun[3,5], Su In Hwang[1], Byung Seok Kim[1], Young Jae Lee[6], Ho Won Jang[2], Jung Ho Yoon[1,6]**

[1]School of Advanced Materials Science and Engineering, Sungkyunkwan University (SKKU), Suwon 16419, Republic of Korea; E-mail: junghoyoon@skku.edu

[2]Department of Materials Science and Engineering, Research Institute of Advanced Materials, Seoul National University, Seoul 08826, Republic of Korea

[3]Electronic and Hybrid Materials Research Center, Korea Institute of Science and Technology (KIST), Seoul 02791, Republic of Korea

[4]Department of Materials Science and Engineering, Korea University, Seoul 02841, Republic of Korea

[5]KU-KIST Graduate School of Converging Science and Technology, Korea University, Seoul 02841, Republic of Korea

[6]Department of Semiconductor Convergence Engineering, Sungkyunkwan University, Suwon 16491, Republic of Korea

*Corresponding author

Table of Contents

Supporting Figure S1 to S16

Supporting Note S1 to S4

Supporting Table S1

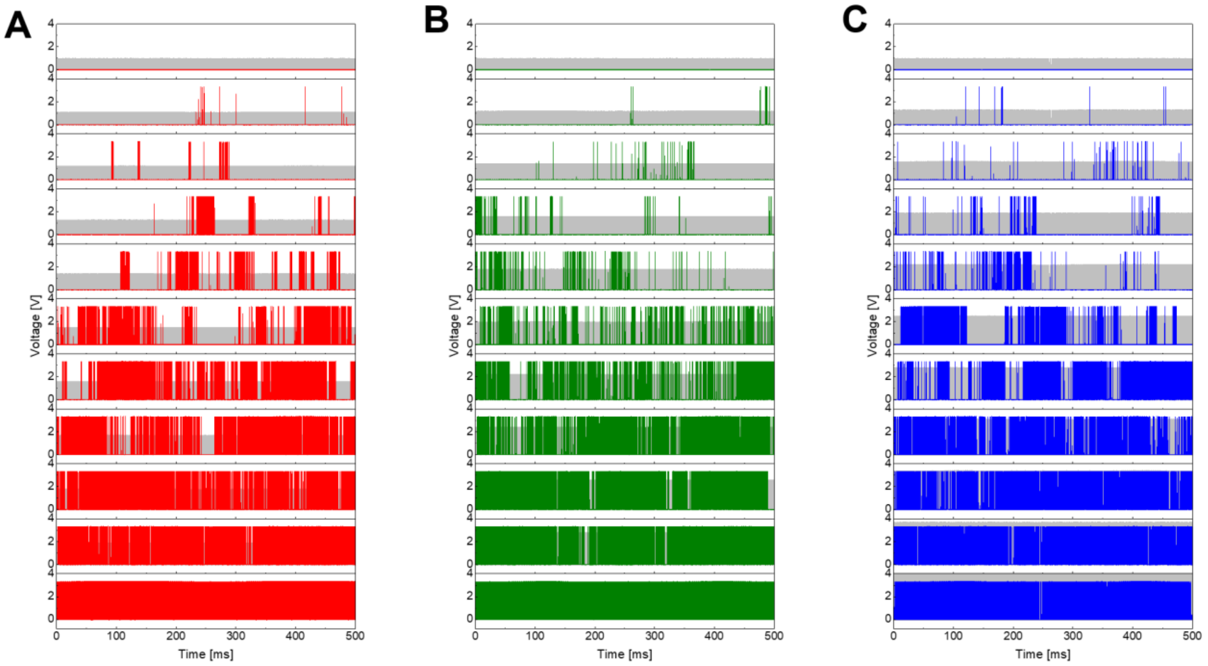

Figure S1. Output probability of the p-bit as a function of input amplitude under (A) 70 µs, (b) 50 µs, and (C) 40 µs pulse width conditions, showing a monotonic increase in switching probability with increasing input amplitude.

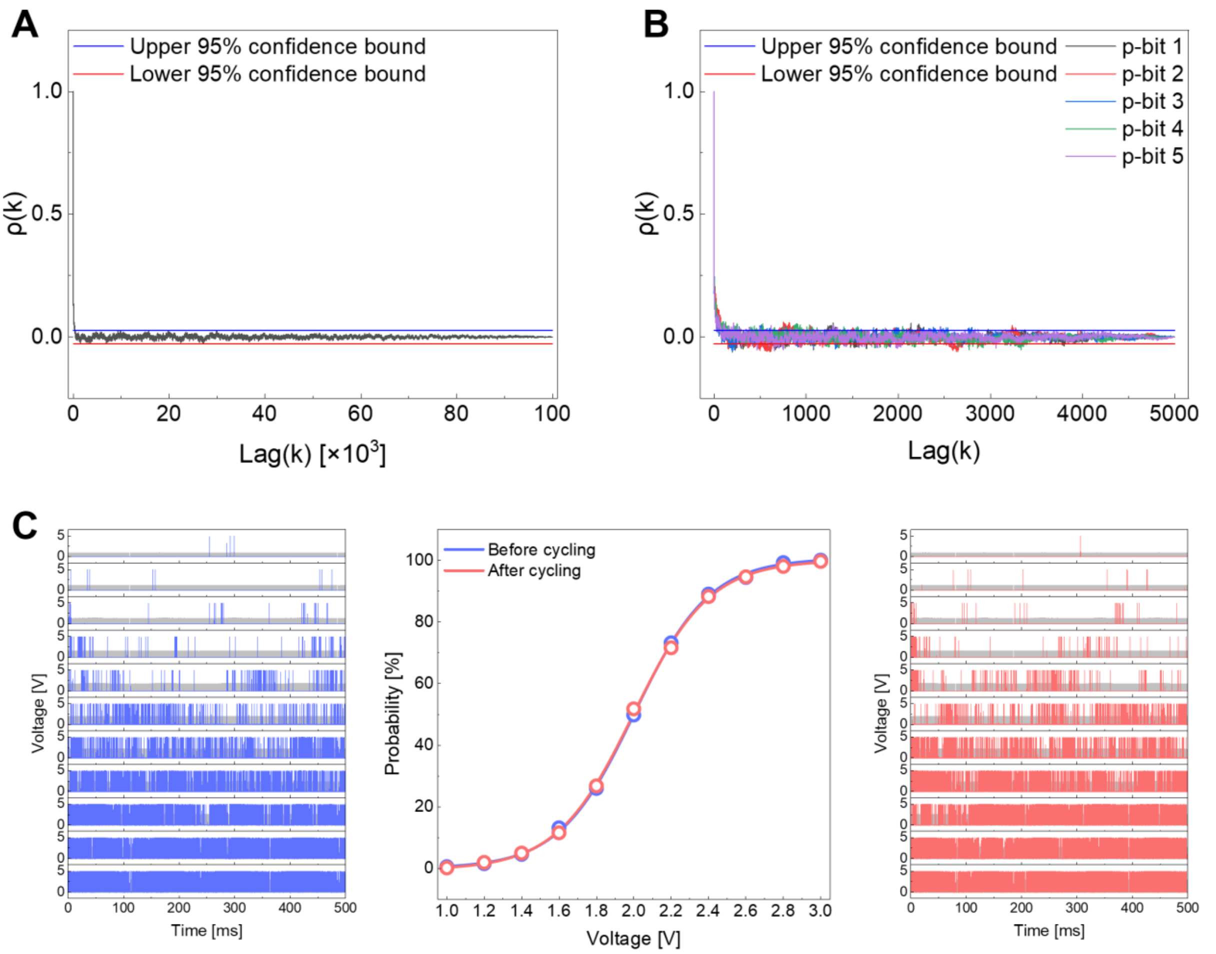

Figure S2. Statistical independence and endurance stability of the volatile memristor-based p-bit. (A) Normalized autocorrelation function calculated from a stochastic bit stream containing 105 bits acquired from a single p-bit operated at an input voltage of 2 V, corresponding to an output probability of approximately 50%. The autocorrelation rapidly decays toward zero with increasing lag, indicating negligible long-range temporal correlation. (B) Normalized autocorrelation functions obtained from five independent p-bits constructed using randomly selected volatile memristors. For each device, a 5 kbit stochastic sequence was acquired under the same operating condition. All p-bits exhibit similar rapid autocorrelation decay behavior, demonstrating reproducible memoryless stochasticity across devices. (C) Endurance evaluation of the volatile memristor-based p-bit under prolonged stochastic operation. Output waveforms measured at varying input amplitudes before cycling (left, blue) and after 105 pulse cycles (right, red), together with the extracted output probabilities and fitted sigmoid characteristics (center).

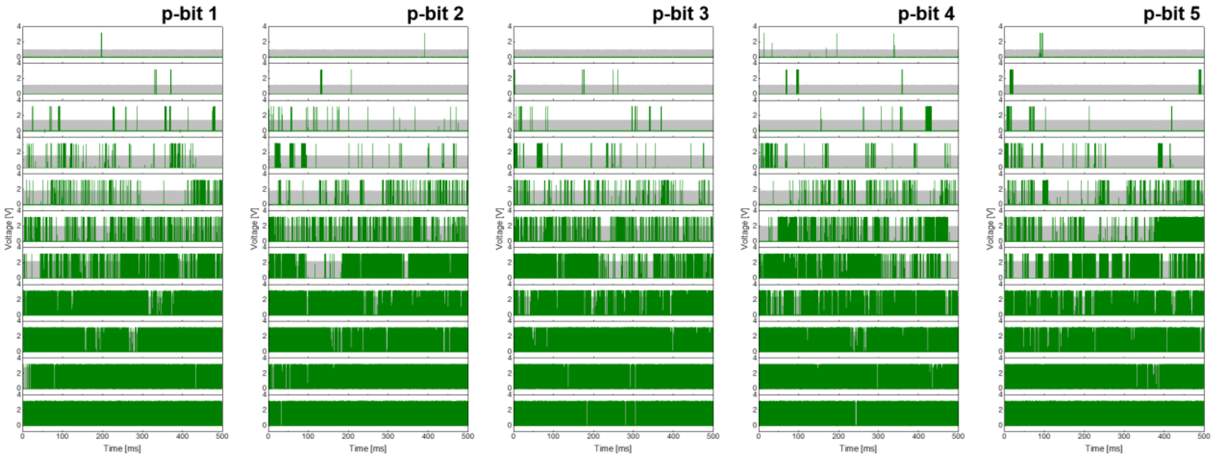

Figure S3. Device-to-device variation of p-bit probability characteristics.

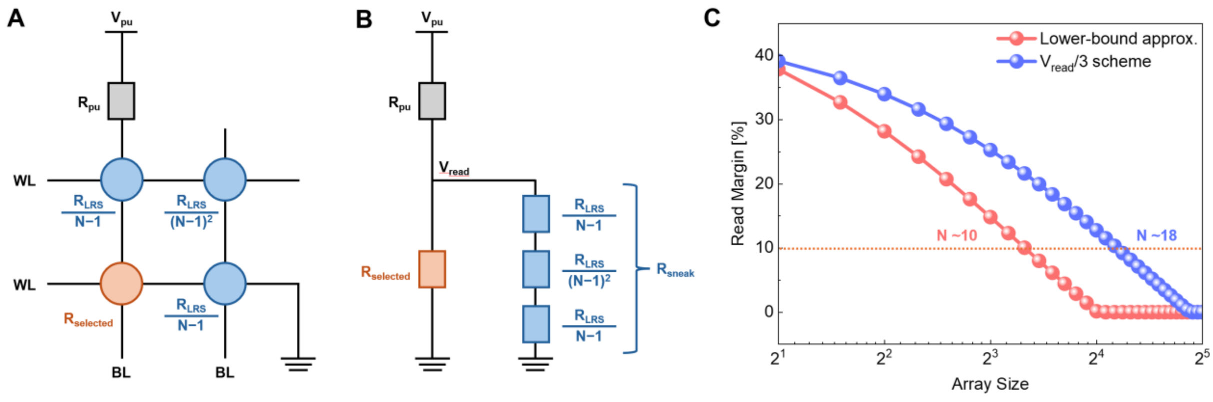

Figure S4. Read margin analysis using the one-bit line pull-up (OBPU) method. (A) Schematic illustration of the worst-case CBA configuration used for read margin evaluation, where the selected cell is in the HRS and all unselected cells are in the LRS. (B) Equivalent sneak path model showing the minimum sneak current path composed of three unselected cells, from which the equivalent sneak path resistance (R_{sneak}) is derived. (C) Calculated read margin as a function of CBA size using the lower-bound R_{sneak} approximation and V_{read}/3 operation schemes. The maximum achievable array size was determined using a 10% read margin criterion.

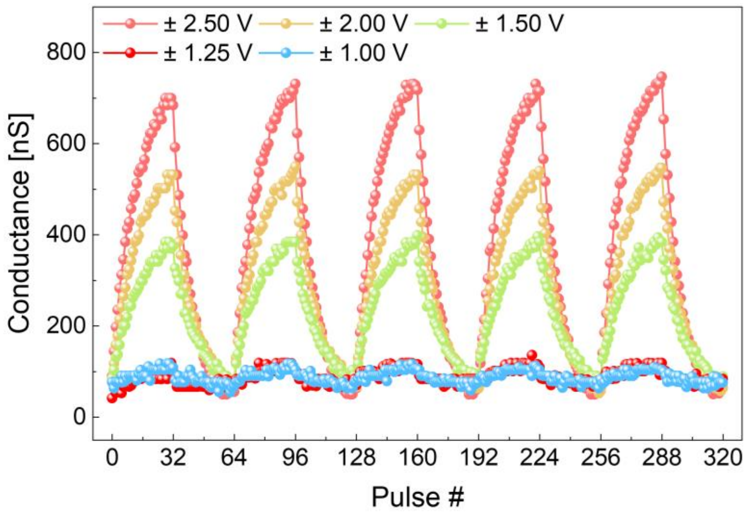

Figure S5. Bias-dependent conductance modulation of a non-volatile memristor under reduced programming voltages. The conductance modulation was performed under voltage pulses of ±2.50 V, ±2.00 V, ±1.50 V, ±1.25 V, and ±1.00 V to evaluate the onset condition for stable weight updating. While pronounced and gradual conductance modulation was observed at ±2.50 V, ±2.00 V, and ±1.50 V, negligible conductance change is detected at ±1.25 V and ±1.00 V under identical pulse conditions.

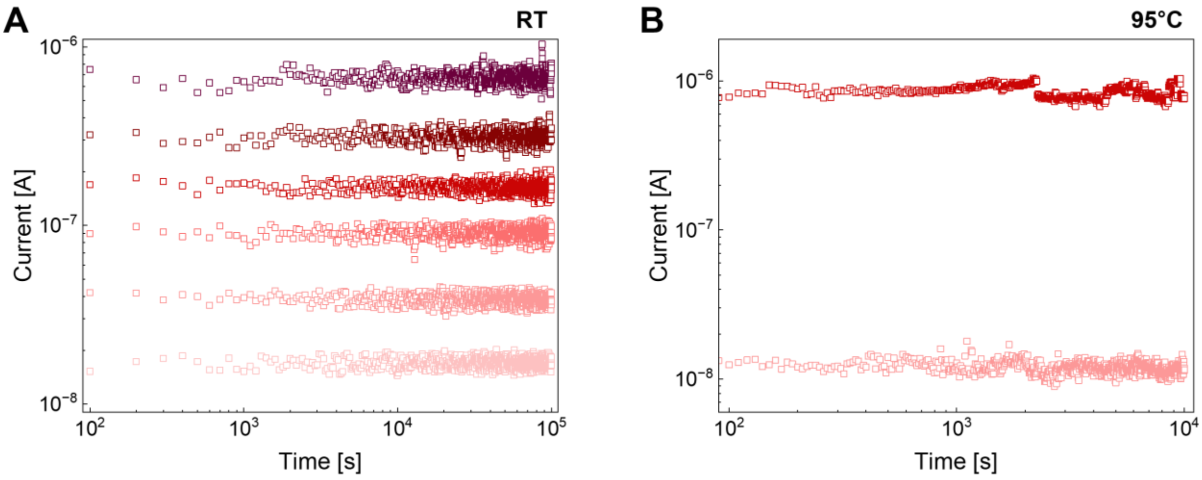

Figure S6. Long-term retention characteristics of the non-volatile memristor used for crossbar weight storage. (A) Retention performance of six programmed intermediate resistance states measured at room temperature for 10^5 s. (B) Accelerated retention test of the HRS and LRS measured at 95 °C for 10^4 s to evaluate the possibility of thermally induced Ru nanocluster migration.

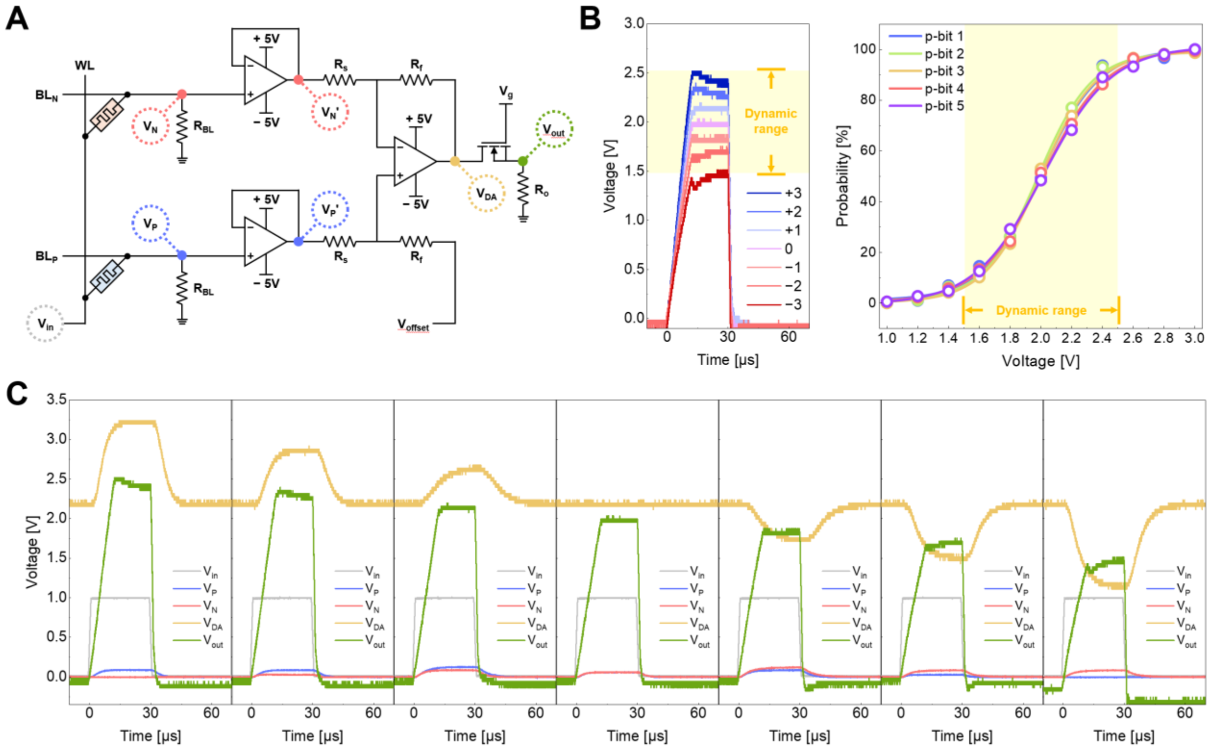

Figure S7. Calibration and signal propagation of the peripheral circuit for mapping CBA-generated local fields to the p-bit operating range. (A) Simplified circuit schematic illustrating a single BL_P, BL_N, and WL together with the associated unity-gain buffers and differential amplifier (DA). Key circuit nodes and resistive components used in the signal processing path are labeled. (B) Comparison between the experimentally calibrated output voltage (V_{out}) and the measured p-bit sigmoid characteristic, showing that the output range is aligned with the effective dynamic range of the p-bit. The corresponding operating windows are highlighted by the yellow shaded region. (C) Monitored voltage evolution through the signal processing chain for local-field values ranging from +3 to −3, including the V_{in}, V_N, V_P, V_{DA}, and V_{out}, demonstrating the preservation of distinguishable voltage levels throughout the calibration process.

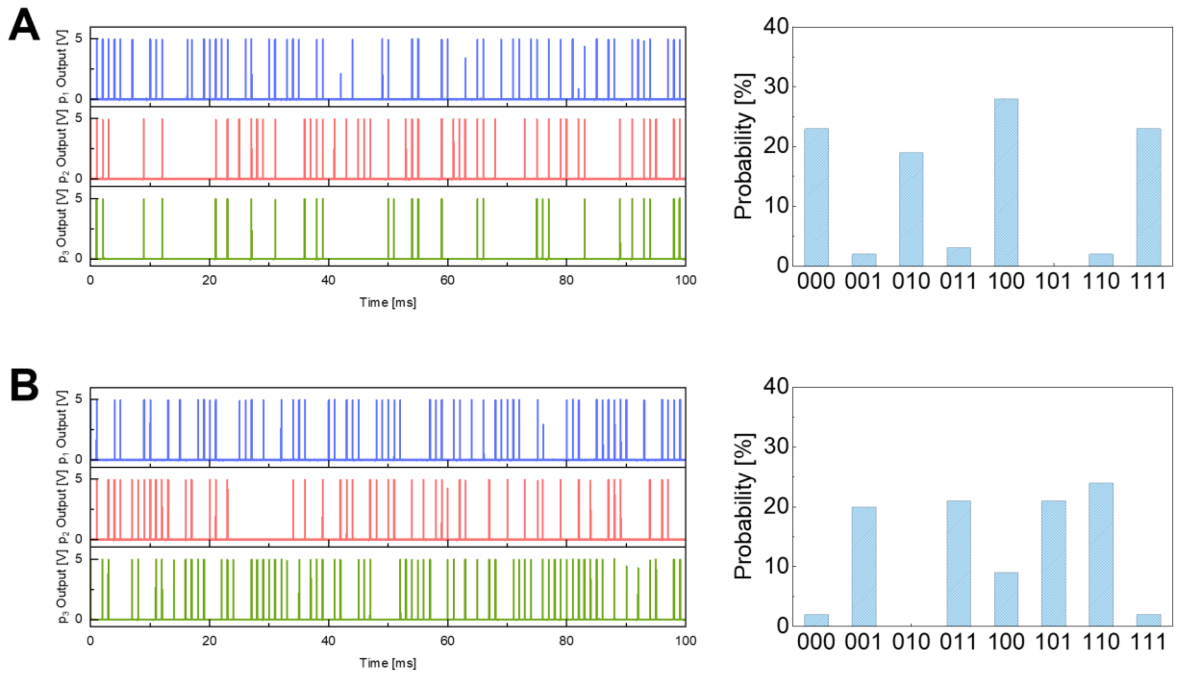

Figure S8. Unconstrained stochastic state distributions of logic gate operations. (A) Distribution of all possible state combinations (000–111) obtained from stochastic iterations of the AND gate without input/output constraints, showing equal probability among valid states. (B) Corresponding unconstrained state distribution for the NAND gate, confirming correct probabilistic behavior.

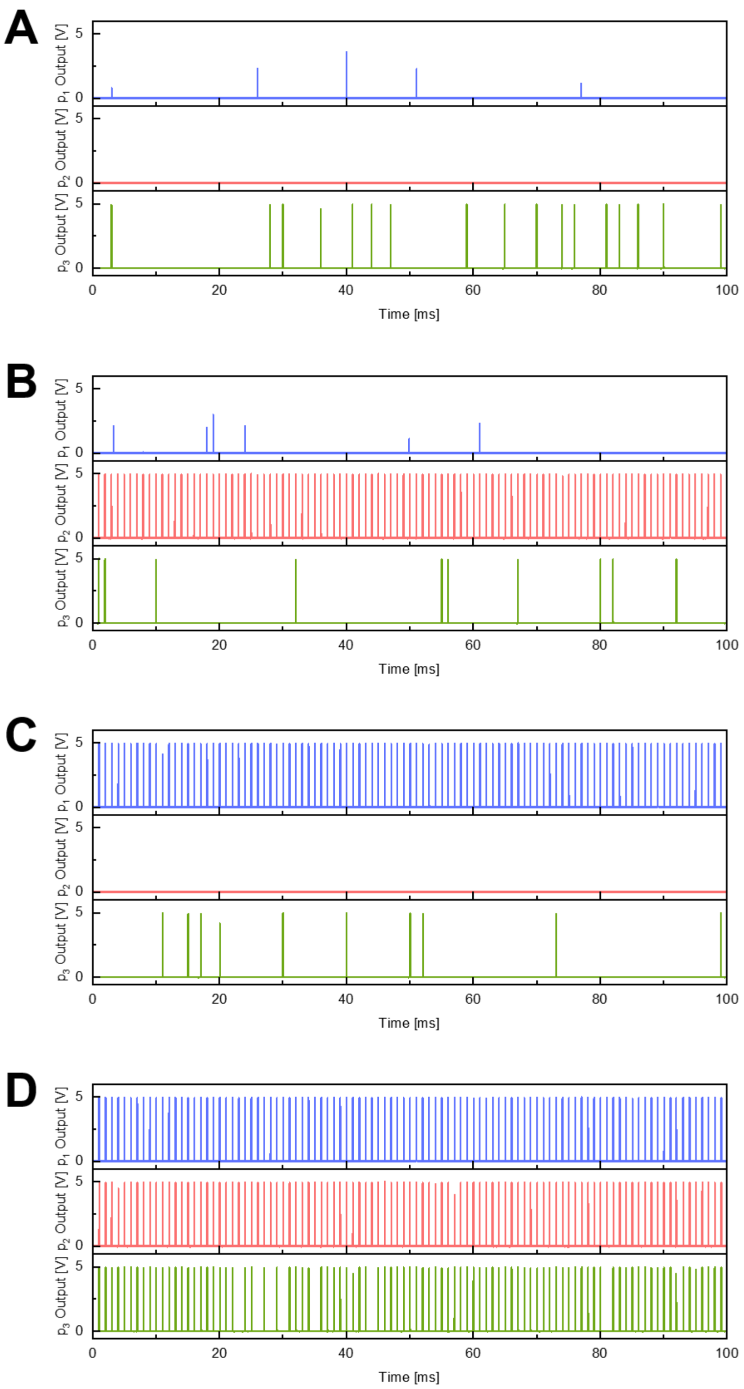

Figure S9. Monitored stochastic output signals for forward AND gate operation, where (p_1, p_2) were fixed at (A) (0, 0), (B) (0, 1), (C) (1, 0), and (D) (1, 1), respectively. The p_3 output predominantly follows the AND logic under each input condition.

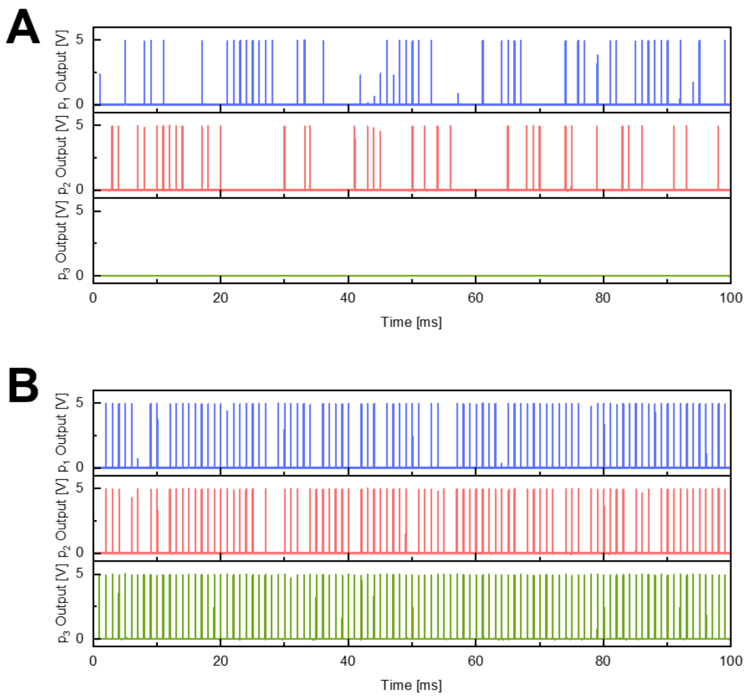

Figure S10. Monitored stochastic output signals for inverse AND gate operation, where p_3 was fixed at (A) 0, (B) 1, respectively. The (p_1, p_2) output predominantly follows the AND logic under each output condition.

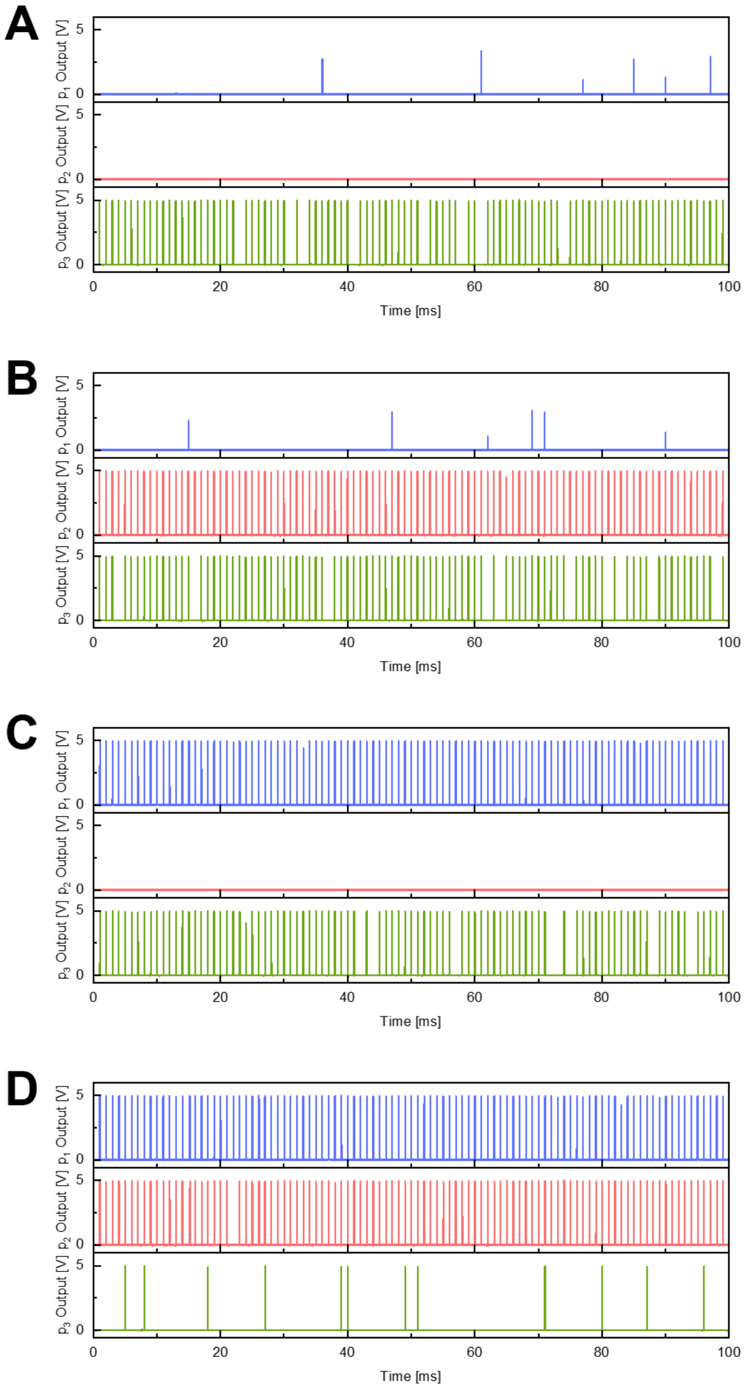

Figure S11. Monitored stochastic output signals for forward NAND gate operation, where (p_1, p_2) were fixed at (A) (0, 0), (B) (0, 1), (C) (1, 0), and (D) (1, 1), respectively. The p_3 output predominantly follows the NAND logic under each input condition.

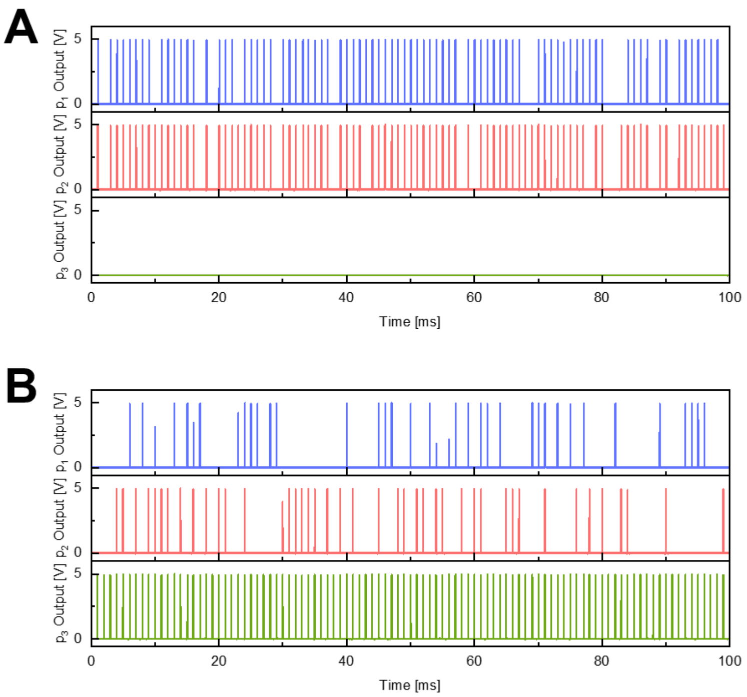

Figure S12. Monitored stochastic output signals for inverse NAND gate operation, where p_3 was fixed at (A) 0, (B) 1, respectively. The (p_1, p_2) output predominantly follows the NAND logic under each output condition.

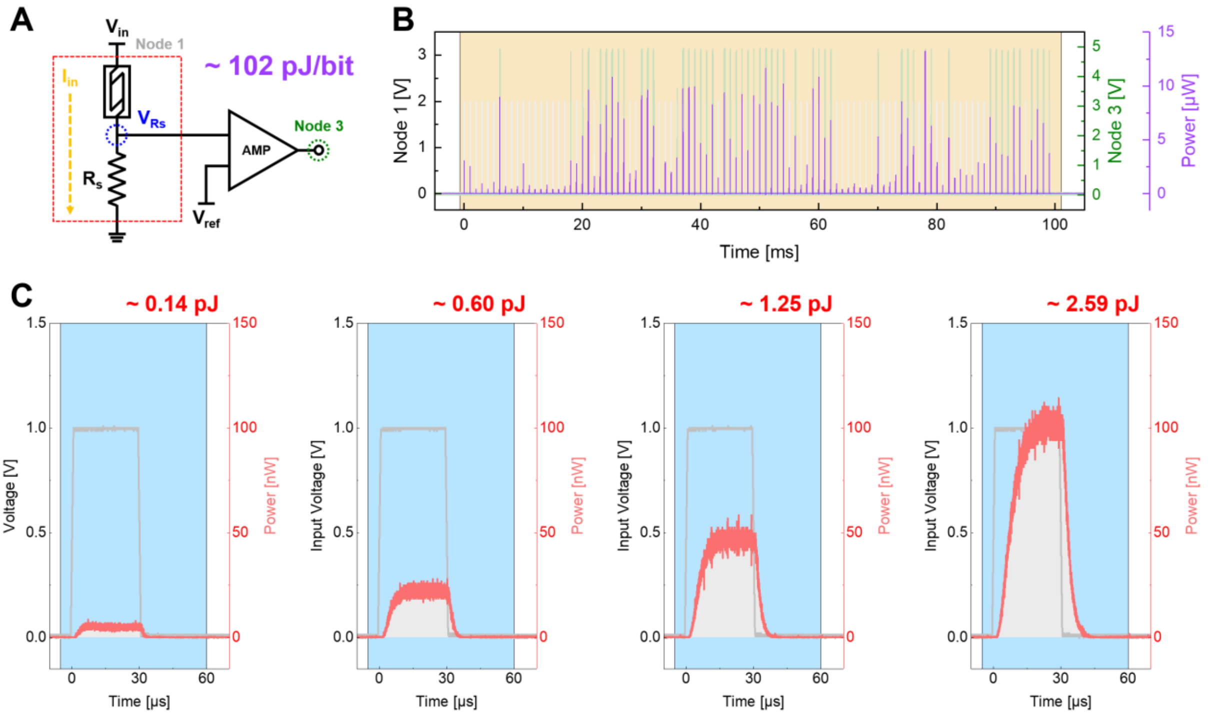

Figure S13. (A) Schematic of the p-bit, where the red dotted box indicates the region used for energy evaluation. (B) Measured V_{in}, p-bit output voltage, and instantaneous power waveforms. The shaded area denotes the integration interval used to calculate the energy consumption per p-bit update. (C) Power consumption profiles used for CBA read energy evaluation. From left to right, the measured instantaneous power waveforms correspond to BL read operations with accumulated weight values of 1, 2, 3, and 4, respectively. In each case, a 1 V read pulse with a width of 30 μs was applied, and the energy consumption was obtained by integrating the instantaneous power over the shaded region, which indicates the integration interval.

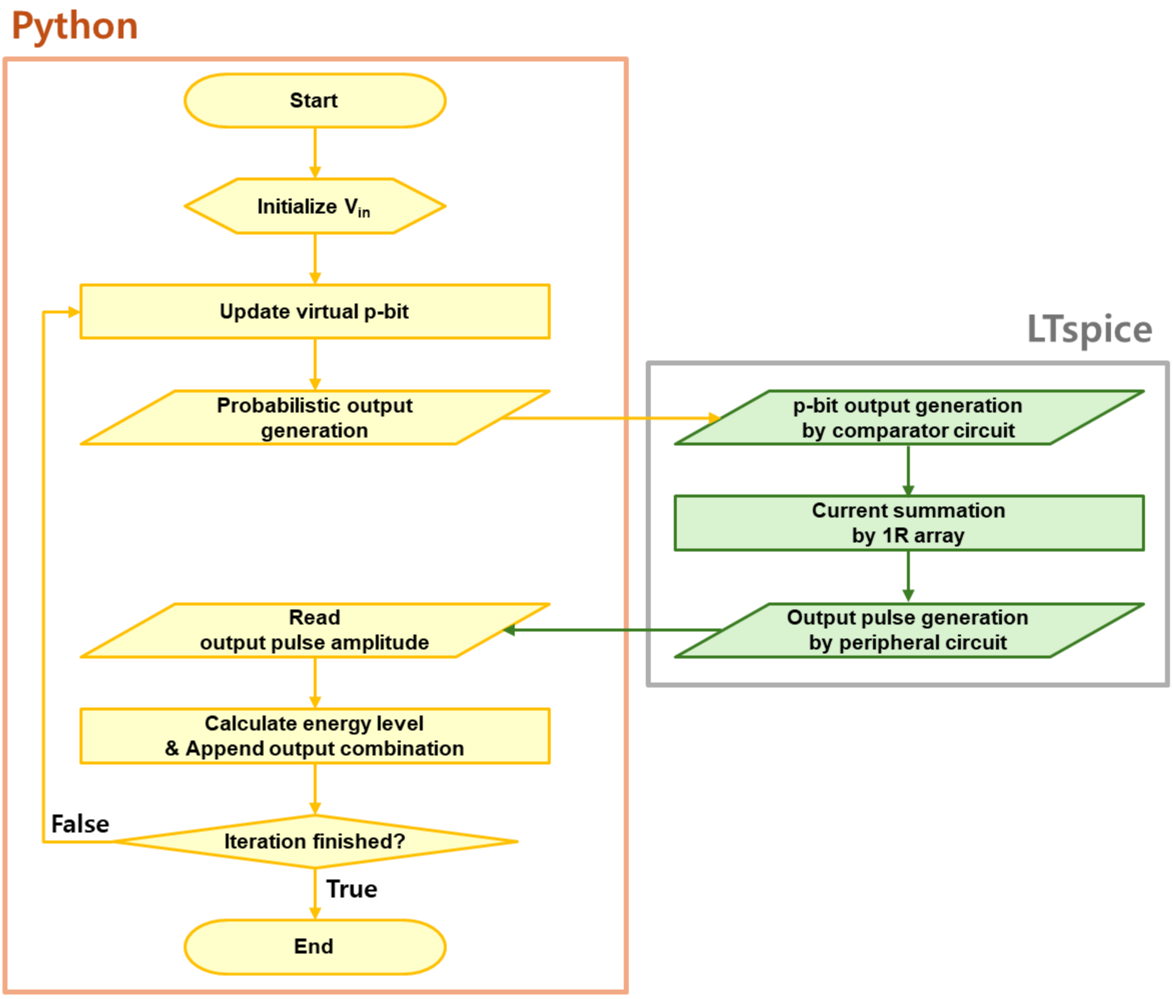

Figure S14. A flowchart of an integrated python environment and LTspice simulator.

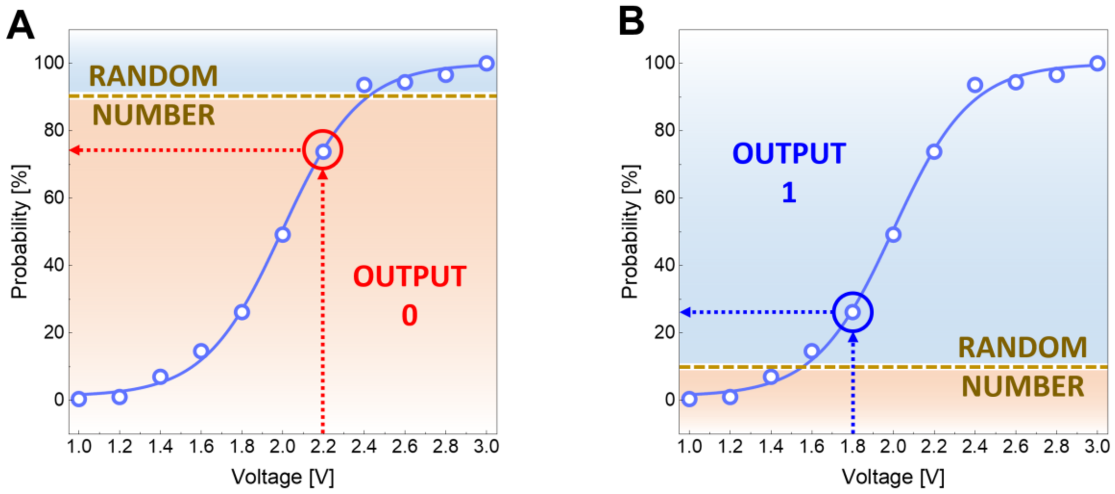

Figure S15. Implementation of the virtual p-bit. Algorithm-level implementation of a virtual p-bit used for system-level simulation, emulating the stochastic sigmoid response of hardware p-bits. The probabilistic output is generated based on a sigmoid curve obtained from a hardware p-bit incorporating a volatile memristor, where the input voltage corresponds to the probability of output "1". For a given input amplitude, the corresponding probability is extracted from the sigmoid curve and compared with a uniformly generated random number. (A) If the random number exceeds the probability, the output is set to 0. (B) If the random number is smaller than the probability, the output is set to 1, thereby reproducing the stochastic behavior of the hardware p-bit.

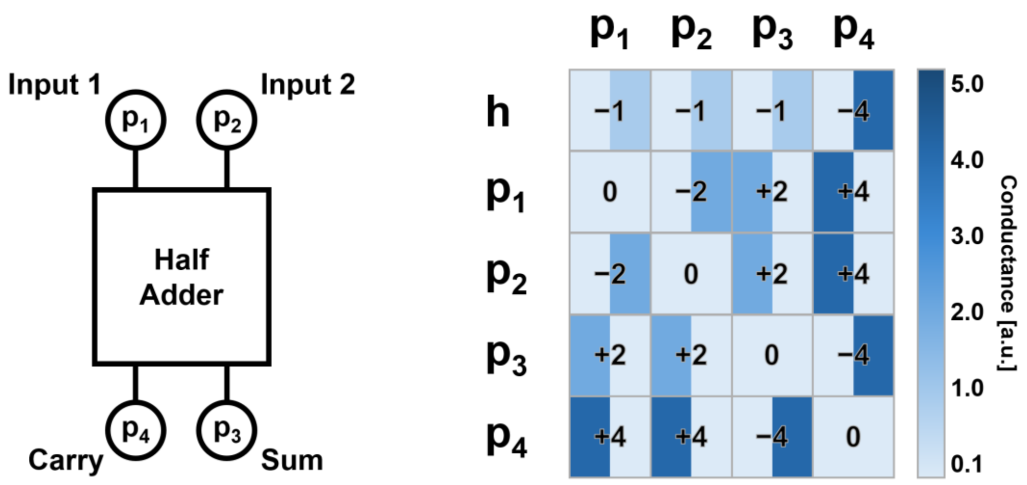

Figure S16. Illustration of p-bit assignment for each node of a half adder, along with the corresponding *J* matrix and *h* vector.

Note S1

To quantitatively evaluate the device-to-device variation of the fabricated p-bits, the input voltages corresponding to a 50% output probability were extracted from the sigmoid curves presented in **Figure 2J** and **Figure S3**. The extracted values were 1.99914 V, 1.98134 V, 1.99420 V, 2.01120 V, and 2.01483 V, yielding an average value of 2.00014 V with a maximum deviation of ±18.8 mV from the mean. This result demonstrates good uniformity of the sigmoid characteristics across the fabricated p-bits.

In the proposed architecture, conductance programming errors, conductance drift, and peripheral-circuit noise do not alter the intrinsic characteristics of the p-bit but instead perturb the effective analog input generated by the CBA and applied to the p-bit. Therefore, their impact can be understood in terms of deviations in the input operating point of the p-bit characteristic.

For the demonstrated AND-gate implementation in **Figure 5A**, the required local-field values span from −3 to +3. By linearly mapping this range onto the p-bit input range of 1.5–2.5 V, adjacent mapped levels are separated by approximately 0.167 V. Consequently, the operating point corresponding to 2.0 V is bounded by neighboring decision boundaries at approximately 1.917 V and 2.083 V, allowing adjacent local-field levels to remain distinguishable under the demonstrated operating conditions. The measured maximum p-bit variation (±18.8 mV in the 50% switching point) is substantially smaller than this level spacing.

Furthermore, the p-bit exhibits a sigmoid characteristic whose sensitivity is highest near the midpoint of the dynamic range and gradually decreases toward both ends as the response approaches saturation. As a result, identical input perturbations introduced near the upper or lower limits of the dynamic range produce smaller changes in the output probability than perturbations near the midpoint.

More generally, the tolerance to conductance programming errors, drift, and peripheral-circuit noise depends on the available input dynamic range, the number of mapped conductance levels, and the target combinatorial optimization problem. The discussion presented here provides a representative interpretation based on the demonstrated AND-gate implementation together with the experimentally measured p-bit characteristics.

Note S2

The read margin was evaluated using the one-bit line pull-up (OBPU) method under the lower-bound sneak path resistance (R_{sneak}) approximation and the $V_{read}/3$ operation schemes. Line resistance was neglected to evaluate the maximum CBA size considering only the effect of sneak path leakage current through unselected cells. To assume the worst-case scenario, all unselected cells were set to low-resistance state (LRS) when the selected cell was in high-resistance state (HRS), whereas all unselected cells were set to HRS when the selected cell was in LRS. The worst-case CBA configuration used for the analysis is illustrated in **Figure S4A**.

When a sneak path current flows in an N × N CBA, it passes through at least three unselected cells: forward through the first and third cells and reverse through the second. The corresponding equivalent sneak path circuit is shown in **Figure S4B**. Within the lower-bound R_{sneak} approximation, the resistance of each element in the representative sneak path was independently selected to maximize its contribution to the sneak current. Consequently, the forward-biased sneak cells were represented by $R_{Vread/2}$, while the reverse-biased sneak cell was represented by R_{-Vread}, yielding a conservative lower-bound estimate of the equivalent R_{sneak}. Accordingly, the equivalent R_{sneak} was approximated as

$$R_{sneak} = \frac{2 \times R_{V_{read}/2}}{N-1} + \frac{R_{-V_{read}}}{(N-1)^2}$$

Under the $V_{read}/3$ operation scheme, the two forward-biased sneak cells were biased at $V_{read}/3$, whereas the reverse-biased sneak cell was biased at $-V_{read}/3$. Accordingly, $R_{Vread/3}$ and $R_{-Vread/3}$ were used for the forward- and reverse-biased sneak cells, respectively. Therefore, the equivalent sneak path resistance was calculated as

$$R_{sneak} = \frac{2 \times R_{V_{read}/3}}{N-1} + \frac{R_{-V_{read}/3}}{(N-1)^2}$$

Where N is the number of WLs and BLs, and $R_{Vread/2}$, R_{-Vread}, $R_{Vread/3}$, and $R_{-Vread/3}$ are the resistance values extracted from the I–V characteristics shown in **Figure 3E**.

The total resistance sensed by the voltage divider was calculated as the equivalent parallel resistance of the selected cell resistance and the sneak path resistance:

$$R_{sense\_LRS} = \frac{R_{selected\_LRS} \times R_{sneak\_HRS}}{R_{selected\_LRS} + R_{sneak\_HRS}}$$

$$R_{sense\_HRS} = \frac{R_{selected\_HRS} \times R_{sneak\_LRS}}{R_{selected\_HRS} + R_{sneak\_LRS}}$$

where $R_{selected\_LRS}$ and $R_{selected\_HRS}$ correspond to the resistances of the selected cell in the LRS and HRS, respectively.

Finally, the read margin was defined as

$$Read\ margin = \frac{\Delta V}{V_{pu}} = \left(\frac{R_{pu}}{R_{sense\_LRS} + R_{pu}}\right) - \left(\frac{R_{pu}}{R_{sense\_HRS} + R_{pu}}\right) \times 100\ (\%)$$

Here, a read margin larger than 10% was considered sufficient to distinguish between the two resistance states. The maximum achievable array size was defined as the largest N × N CBA satisfying the 10% read margin criterion. The calculated read margins as a function of CBA size are summarized in **Figure S4C**. Using the OBPU method with the lower-bound R_{sneak} approximation, a 10 × 10 crossbar array size satisfying the 10% read margin criterion was obtained. By comparison, employing the $V_{read}/3$ operation scheme increased the achievable array size to 18 × 18.

Note S3

We derive a quadratic energy function that encodes the logical relation of a two-input AND gate, $p_3 = p_1 p_2$, using binary variables $p_1, p_2, p_3 \in \{0,1\}$. Rather than introducing a predefined quadratic unconstrained binary optimization (QUBO) form, we begin with the most general quadratic energy function over three binary variables and determine its coefficients by enforcing ground-state conditions corresponding to the truth table of the AND operation. The general quadratic energy function can be written as

$$E(p_1, p_2, p_3) = h_1 p_1 + h_2 p_2 + h_3 p_3 + J_{12} p_1 p_2 + J_{13} p_1 p_3 + J_{23} p_2 p_3,$$

where h_i and J_{ij} are unknown coefficients to be determined. The objective is to assign these coefficients such that the valid logical configurations satisfying $p_3 = p_1 p_2$ correspond to the global minima of the energy landscape. From the truth table of the AND gate, the valid configurations are $(p_1, p_2, p_3) = (0,0,0), (0,1,0), (1,0,0), (1,1,1)$. We impose that these configurations have equal minimum energy, which we set to zero without loss of generality. Substituting these configurations into the energy function yields the following constraints:

$$E(0,0,0) = 0,$$
$$E(0,1,0) = h_2 = 0,$$
$$E(1,0,0) = h_1 = 0,$$
$$E(1,1,1) = h_1 + h_2 + h_3 + J_{12} + J_{13} + J_{23} = 0.$$

From these conditions, we obtain $h_1 = h_2 = 0$, and the remaining constraint reduces to

$$h_3 + J_{12} + J_{13} + J_{23} = 0.$$

Next, we enforce that all invalid configurations have strictly positive energy. For the configuration $(1,1,0)$, which violates the AND relation, we obtain

$$E(1,1,0) = J_{12} > 0,$$

which requires $J_{12} > 0$. For $(0,0,1)$,

$$E(0,0,1) = h_3 > 0,$$

implying $h_3 > 0$. For the configurations $(1,0,1)$ and $(0,1,1)$, we obtain

$$E(1,0,1) = h_3 + J_{13} > 0,$$
$$E(0,1,1) = h_3 + J_{23} > 0,$$

which impose additional inequalities on J_{13} and J_{23}.

A simple choice of coefficients satisfying all these constraints is obtained by selecting $J_{12} = 1$, $h_3 = 3$, and $J_{13} = J_{23} = -2$, which also satisfies the equality condition for the valid configuration $(1,1,1)$. Substituting these values yields the quadratic energy function

$$E_{\mathrm{AND}}(p_1, p_2, p_3) = p_1 p_2 - 2 p_1 p_3 - 2 p_2 p_3 + 3 p_3.$$

This construction ensures that all valid configurations corresponding to the AND operation have zero energy, while all invalid configurations are assigned positive energy, thereby correctly encoding the logic in the ground-state manifold. The resulting expression contains only linear and pairwise interaction terms and is therefore directly compatible with the QUBO framework.

The same framework can be extended to derive the QUBO representation of a NAND gate, whose logical relation is given by $p_3 = \overline{p_1 p_2}$. Using the identical general quadratic energy function,

$$E(p_1, p_2, p_3) = h_1 p_1 + h_2 p_2 + h_3 p_3 + J_{12} p_1 p_2 + J_{13} p_1 p_3 + J_{23} p_2 p_3,$$

the valid NAND configurations are $(p_1, p_2, p_3) = (0,0,1), (0,1,1), (1,0,1), (1,1,0)$. One key difference from the AND gate case is that directly assigning zero energy to all valid NAND configurations does not properly separate the valid and invalid states. In particular, the invalid configuration $(0,0,0)$ inherently satisfies

$$E(0,0,0) = 0,$$

because all linear and quadratic terms simultaneously vanish. Consequently, the valid NAND configurations must possess energies lower than that of the $(0,0,0)$ state in order to ensure that the correct logical states correspond to the ground states of the energy landscape. Therefore, instead of constraining the valid logical states to zero energy, the coefficients should be selected such that all valid NAND states possess lowest energy while the invalid states remain energetically unfavorable. Thus, substituting valid and invalid configurations into the energy function yields the following constraints:

$$E(0,0,0) = 0,$$
$$E(0,0,1) = h_3 < 0,$$
$$E(1,0,0) = h_1 > h_3,$$
$$E(0,1,0) = h_2 > h_3,$$
$$E(0,1,1) = h_2 + h_3 + J_{23} < 0,$$
$$E(1,0,1) = h_1 + h_3 + J_{13} < 0,$$
$$E(1,1,0) = h_1 + h_2 + J_{12} < 0,$$
$$E(1,1,1) = h_1 + h_2 + h_3 + J_{12} + J_{13} + J_{23} > h_3 \rightarrow h_1 + h_2 + J_{12} + J_{13} + J_{23} > 0.$$

These constraints enforce that all valid configurations have strictly negative energy. Furthermore, to ensure that all valid NAND states belong to the same degenerate ground-state manifold, the energies of all valid logical states are constrained to be identical:

$$E(0,0,1) = E(0,1,1) = E(1,0,1) = E(1,1,0).$$

Substituting the corresponding energy expressions yields

$$h_3 = h_2 + h_3 + J_{23} = h_1 + h_3 + J_{13} = h_1 + h_2 + J_{12}.$$

These relations reduce to

$$J_{23} = -h_2,$$
$$J_{13} = -h_1,$$
$$J_{12} = h_3 - h_1 - h_2 > 0.$$
$$J_{12} > 0.$$

A simple choice of coefficients satisfying all these constraints is obtained by selecting $J_{12} = 1$, $J_{13} = J_{23} = -h_1 = -h_2 = 2$, and $h_3 = -3$. Substituting these values yields the NAND energy function

$$E_{\mathrm{NAND}} = p_1p_2 + 2p_1p_3 + 2p_2p_3 - 2p_1 - 2p_2 - 3p_3.$$

Lastly, a QUBO energy function for the half adder is derived. The half adder consists of two binary inputs $p_1$and p_2, and two outputs corresponding to the sum bit $p_3$and carry bit p_4. The valid half-adder configurations are given by $(p_1, p_2, p_3, p_4) = (0,0,0,0), (0,1,1,0), (1,0,1,0), (1,1,0,0)$. These configurations satisfy the linear relation

$$p_1 + p_2 - p_3 - 2p_4 = 0.$$

Since this relation contains only linear terms, constructing the energy function as a squared constraint provides a simple and efficient approach. Furthermore, because the square of a real-valued function is always non-negative, the minimum energy is naturally obtained when the constraint is exactly satisfied. Therefore, the valid logical configurations automatically correspond to the ground states of the energy landscape.

Accordingly, the half-adder energy function is constructed as

$$E_{\mathrm{HA}} = (p_1 + p_2 - p_3 - 2p_4)^2.$$

Expanding the expression gives

$$E_{\mathrm{HA}} = p_1^2 + p_2^2 + p_3^2 + 4p_4^2 + 2p_1p_2 - 2p_1p_3 - 2p_2p_3 - 4p_1p_4 - 4p_2p_4 + 4p_3p_4.$$

Using the binary property $p_i^2 = p_i$, the quadratic energy function becomes

$$E_{\mathrm{HA}} = p_1 + p_2 + p_3 + 4p_4 + 2p_1p_2 - 2p_1p_3 - 2p_2p_3 - 4p_1p_4 - 4p_2p_4 + 4p_3p_4.$$

This formulation guarantees that all valid half-adder configurations yield the minimum energy of zero, while invalid configurations are assigned positive energy penalties.

In practical implementations using 1R CBA, the state update dynamics are governed by the local field obtained from the partial derivative of the energy with respect to each variable. Since the next state is typically determined by the sign of the negative gradient, i.e., $-\partial E/\partial p_i$, the effective coupling coefficients used in the hardware correspond to the negative of those appearing in the energy function. Therefore, when mapping the QUBO coefficients onto physical conductance, the overall sign of the coefficients is inverted during programming to ensure that the system evolves toward the intended ground state.

Note S4

To ensure reliable probabilistic operation, the analog weighted-sum values generated by the CBA were calibrated to the experimentally characterized input range of the p-bit through the peripheral signal-conditioning circuitry. **Figure S7A** illustrates the simplified schematic of the calibration path, including representative BL_P, BL_N, and WL nodes, unity-gain buffers, the differential amplifier (DA), and the NMOS switching stage.

Within the CBA, conductance-weighted currents are accumulated on the BLs to generate the local field. Depending on the combination of the *J* matrix and *h* vector, intermediate BL nodes may exhibit accumulated weights exceeding the final local-field values. For example, the BL_{3P} and BL_{3N} nodes can each reach a maximum accumulated weight of +4 before differential processing. After subtraction in the DA, however, the effective local field delivered to the p-bit spans the intended integer range from −3 to +3 for the demonstrated AND and NAND gate implementations.

To achieve uniform voltage spacing between adjacent local-field levels, the peripheral circuit was calibrated such that integer weights of 1, 2, 3, and 4 produced approximately 30, 60, 90, and 120 mV across the BL pull-down resistor (R_{BL}), respectively. These voltages were buffered by unity-gain OP-AMPs and subsequently amplified by the DA according to

$$V_{DA} = (V_P' - V_N')\left(\frac{R_f}{R_s}\right) + V_{offset}$$

where R_s and R_f denote the input and feedback resistors, respectively. The resistor values were selected as R_s = 30 kΩ and R_f = 360 kΩ, corresponding to a gain of 12. An offset voltage (V_{offset}) of 2.2 V was employed to compensate for practical loading effects in the downstream circuitry and to align the resulting signal range with the experimentally measured operating window of the p-bit. Consequently, the DA output spans approximately 1.2–3.2 V.

The DA output is subsequently applied to the drain of an NMOS switching transistor, where the voltage sampled across the source-side resistor R_o is aquired at the microcontroller. Owing to practical non-idealities in the switching stage and interactions with the downstream circuitry, the measured voltage across R_o occupies approximately 1.5–2.5 V, as shown in **Figure S7B**. This operating range remains well aligned with the probability transition region of the experimentally measured sigmoid characteristic, ensuring that different local-field values are mapped to distinguishable output probabilities without excessive overlap or saturation.

The transient voltage evolution throughout the signal-processing chain is summarized in **Figure S7C** for local-field values ranging from −3 to +3. Although finite rise and fall times are observed due to parasitic capacitances and the limited bandwidth of the analog peripheral circuits, the relative separation between adjacent local-field levels is preserved throughout the propagation from V_{in} to V_N, V_P, V_{DA}, and finally V_{out}. Therefore, the dominant effect of the RC-limited dynamics is a common temporal delay rather than degradation of the weighted-sum representation.

These results indicate that, under the operating conditions used in this work, the practical non-idealities of the peripheral circuitry do not compromise the distinguishability of the analog weighted-sum levels or the probabilistic operation of the p-bit. Further optimization of the differential amplifier and switching stage is expected to reduce transient distortion and improve voltage transfer fidelity.

	Device structure	Energy/Update	CBA read energy	Total system-level energy	Ref.
CMOS	n-p-n bipolar junction	58 pJ	Not implemented	-	[S1]
	Floating body MOSFET	1.1 fJ	Not implemented	-	[S2]
	n+ poly-Si/SiO_2/SiN_4/Si	125 pJ	1.49 pJ	10.03 nJ	[S3]
MTJ	Ta/Pt/$[Co/Pt]_7$/Co/Ru/ $[Co/Pt]_2$/Co/Ta/CoFeB/ MgO/CoFeB/Ta/Ru/Ta	2 fJ	Not implemented	-	[S4]
FTJ	Pt/$Hf_{0.5}Zr_{0.5}O_2$/TiO_2/TiN	151.2 fJ	0.36 pJ	2.1 nJ	[S5]
Memristor	$Cu_{0.1}Te_{0.9}$/HfO_2/Pt	23 pJ	Not implemented	-	[S6]
	Pt/TiN/NbO_x/TiN-via/Pt	128 pJ	Not implemented	-	[S7]
	Au/SiO_x NRs/Pt	4.06 pJ	Not implemented	-	[S8]
	Pd/$NdNiO_3$/Au	110 pJ	Not implemented	-	[S9]
	Pt/SiO_2 NRs/Ag/Pt (This work)	102 pJ	0.94 pJ	31.16 nJ	

Table S1. Quantitative comparison of the estimated energy consumption of the proposed architecture with previously reported CMOS-, MTJ-, FTJ-, and memristor-based probabilistic computing implementations. The table summarizes the p-bit energy per update, crossbar read energy, and total system-level energy, where available, under the respective evaluation methodologies reported in each work.

References

[S1] J. Kim, J.-K. Han, H.-Y. Maeng, et al.," Fully CMOS-Based p-Bits with a Bistable Resistor for Probabilistic Computing," *Advanced Functional Materials* **2024**, *34* (22), 2307935, https://doi.org/https://doi.org/10.1002/adfm.202307935.

[S2] H.-Y. Maeng, J.-K. Han, J. Han, et al.," Homeothermic P-Bit Computing Hardware with Stochastic Operations Beyond Limit of Non-Stochastic Materials," *Advanced Functional Materials* **2025**, *35* (12), 2417552, https://doi.org/https://doi.org/10.1002/adfm.202417552.

[S3] J.-Y. Park, J.-H. Lee, J.-M. Lee, et al.," CMOS compatible probabilistic computing hardware with cointegrated reconfigurable p-bits and synapse arrays," *Nature Communications* **2026**, *17* (1), 5110, https://doi.org/10.1038/s41467-026-71906-x.

[S4] W. A. Borders, A. Z. Pervaiz, S. Fukami, et al.," Integer factorization using stochastic magnetic tunnel junctions," *Nature* **2019**, *573* (7774), 390, https://doi.org/10.1038/s41586-019-1557-9.

[S5] Z. Guan, H. Zhu, Y. Li, et al.," Probabilistic computing utilizing HfO2-based stochastic ferroelectric tunnel junctions," *Nature Communications* **2026**, https://doi.org/10.1038/s41467-026-72742-9.

[S6] K. S. Woo, J. Kim, J. Han, et al.," Probabilistic computing using Cu0.1Te0.9/HfO2/Pt diffusive memristors," *Nature Communications* **2022**, *13* (1), 5762, https://doi.org/10.1038/s41467-022-33455-x.

[S7] H. Rhee, G. Kim, H. Song, et al.," Probabilistic computing with NbOx metal-insulator transition-based self-oscillatory pbit," *Nature Communications* **2023**, *14* (1), 7199, https://doi.org/10.1038/s41467-023-43085-6.

[S8] S. Choi, G. S. Kim, J. Yang, et al.," Controllable SiOx Nanorod Memristive Neuron for Probabilistic Bayesian Inference," *Advanced Materials* **2022**, *34* (1), 2104598, https://doi.org/https://doi.org/10.1002/adma.202104598.

[S9] T. J. Park, K. Selcuk, H.-T. Zhang, et al.," Efficient Probabilistic Computing with Stochastic Perovskite Nickelates," *Nano Letters* **2022**, *22* (21), 8654, https://doi.org/10.1021/acs.nanolett.2c03223.
